# Optimization-driven steganographic system based on fused maps and blowfish encryption

**DOI:** 10.1038/s41598-026-35556-9

**Published:** 2026-01-09

**Authors:** Ahmed Aljughaiman, Rana Alrawashdeh

**Affiliations:** 1 Department of Computer Networks and Communications, College of Computer Sciences and Information Technology, King Faisal University, Al-Ahsa, 31982 Saudi Arabia; 2 Department of Information and Computer Science, College of Computing and Mathematics, King Fahd University of Petroleum and Minerals, Dhahran, 31261 Saudi Arabia

**Keywords:** Blowfish, Fused Maps, Particle Swarm Optimization (PSO), Steganography, Engineering, Mathematics and computing

## Abstract

Steganography as a practice is highly useful as it allows information to be concealed within digital images while maintaining visual quality. However, it can be a challenge to balance imperceptibility, capacity, and robustness while ensuring that embedded data remains secure against statistical and Deep Learning (DL)–based steganalysis. Therefore, we propose a new framework that utilizes the BOSSbase dataset for cover images, the USC-SIPI dataset for secreted images, and introduces adaptive image steganography, which combines fused map-based embedding with evolutionary optimization to solve existing problems. The system starts by encrypting the secret image through Blowfish cipher encryption to protect its confidentiality. The model generates a fused map through a combination of entropy and Laplacian-based noise maps from the cover image to determine the most suitable embedding areas. Next, we optimize the process of embedding location selection and ordering using Particle Swarm Optimization (PSO) to maximize both security and image quality. Next, we use priority-guided Least Significant Bits (LSB) substitution in the embedding process to generate an optimized priority map for inserting the encrypted bitstream into the image. The system can function on different cover image sizes, so at $512\times 512$ the resolution is 51-61 dB Peak Signal-to-Noise Ratio (PSNR) and 0.9972-1.0 Structural Similarity Index Measure (SSIM) for stego images at 0.1-1.0 Bits Per Pixel (BPP) with 262,144 bits while maintaining perfect secret reconstruction (SSIM = 1.000) and processing times under 0.25 seconds for embedding and extraction. Security tests demonstrate that the proposed system remains undetectable since the Area Under Curve (AUC) for $\text {Ye-Net} \,/\, \text {Xu-Net}$ reaches 0.49-0.57 across all payload levels and Regular Singular (RS) statistics remain at 0.59-0.66 between 0.1-1.0 BPP. The proposed model provides effective capacity while maintaining high visual quality and security against DL-based steganalysis, such as that using Convolutional Neural Networks (CNN).

## Introduction

The practice of concealing secret information inside digital images through steganography requires both artistic and scientific expertise to ensure the hidden data remains undetected^1,2^. Secure communication heavily relies on image steganography as it enables complete avoidance of hidden content detection^3,4^. The digital arena requires secure data transmission of sensitive information, particularly when the existence of hidden communication needs to stay undisclosed. Image steganography functions as a practical solution through information hiding by embedding secret data into digital images without changing their visual appearance^5,6^. The LSB method stands as a widely used steganographic technique because it provides both ease of use and high capacity for embedding data^7,8^. The traditional LSB techniques face two main challenges as they are vulnerable to statistical steganalysis, they have fixed embedding patterns, limited robustness, and low adaptivity to image content^9,10^.

**Fig. 1:**
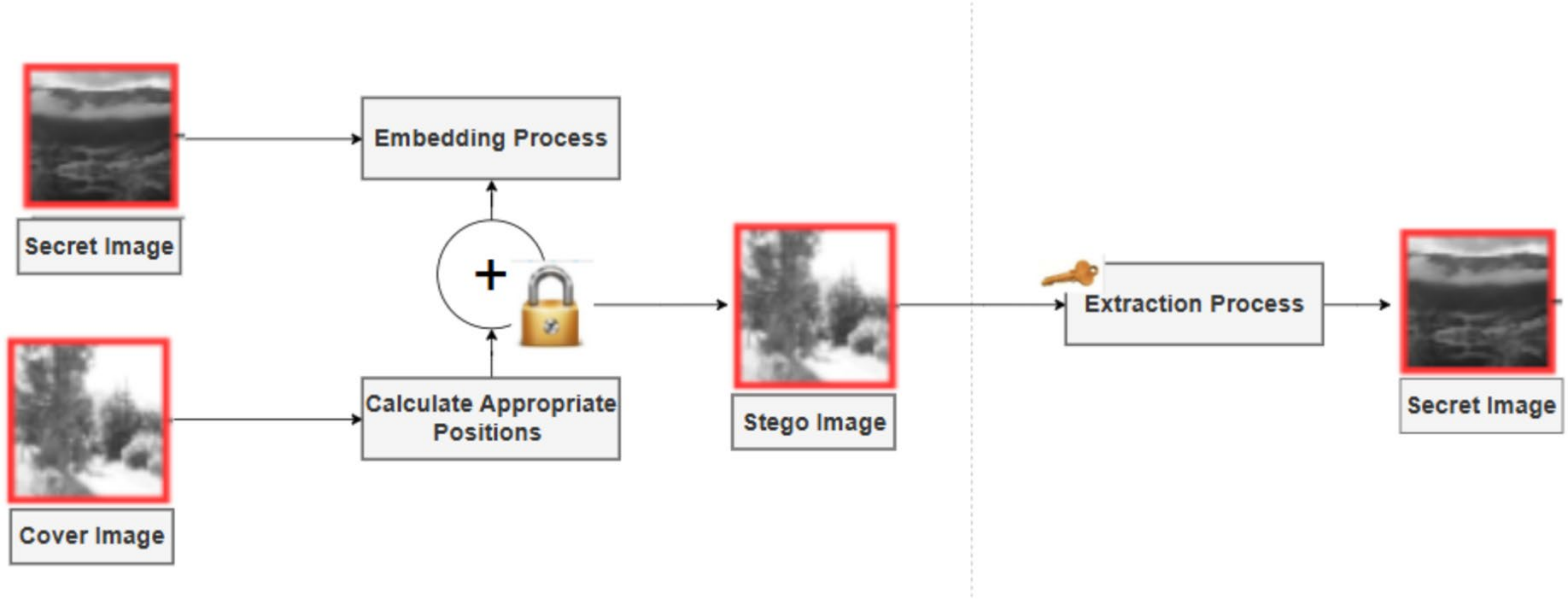
The basic image steganography pipeline. Images were obtained from the open-source BOSSBase dataset and used for academic research purposes.

Traditional image-steganography, as shown in Fig. 1, functions with an embedding process at the sender end and an extraction process on the receiver side. The system begins with two initial inputs, which consist of a cover image and a secret image. The position-selection module examines the cover image to find appropriate spots for embedding data, which tend to be located in rich areas because such regions allow for minimal perceptible changes. The embedding process adds secret information to choose positions in the stego image after the secret image undergoes optional key-based encryption and permutation. At the receiving end, the extraction process utilizes a shared key to select the locations from which to extract the hidden data, allowing for recovery of the secret image^11^.

To address certain limitations of traditional steganography methods, researchers have investigated the use of adaptive and intelligent embedding strategies that use cover image characteristics to address these limitations^12,13^. The identification of high-information areas through local entropy and image gradient measures enables the selection of regions that can withstand modification without significant visual degradation^14,15^. In our work, the proposed system relies on entropy and noise-based maps together with PSO-driven optimization to enhance both imperceptibility and robustness in image steganography. The Blowfish cipher encrypts the secret image before embedding to protect it from image attacks. Next, a fused map is computed from the cover image by blending local entropy and Laplacian-based noise measures, effectively locating texture-rich and unpredictable regions. PSO is then used to optimize the selection and ordering of embedding positions based on a fitness function that jointly maximizes the PSNR and minimizes the Bit Error Rate (BER) during recovery. The objectives of this proposed scheme are as follow:

1. To protect the confidentiality of the secret image through symmetric encryption (Blowfish cipher) before embedding.
2. To generate an adaptive fused map through the combination of entropy and noise characteristics of the cover image to determine optimal embedding locations for imperceptible and robust data hiding.
3. To use PSO to select optimal pixel positions for embedding through a fitness function that optimizes PSNR and BER metrics.
4. To obtain benefit from the combination of a fused map and PSO optimization to enhance adaptability and steganalysis resistance.
5. To evaluate the system through quantitative metrics, including PSNR, SSIM, BER, Mean Squared Error (MSE), Image Fidelity (IF), BPP, and execution time (embedding and extraction time), to assess its performance in terms of imperceptibility, robustness, capacity, and processing time.

This work contributes to the field by introducing a new image steganography system that integrates three essential improvements to boost security and imperceptibility. The system uses an entropy and noise-based fused map to direct data embedding operations toward areas that are less sensitive to human visual perception. The second improvement uses PSO to determine pixel embedding sequences through a fitness function that maintains both visual quality (PSNR) and recovery accuracy (BER). The system implements Blowfish encryption before embedding to guarantee complete content confidentiality. The proposed model combines these components into a unified adaptive system that produces superior robustness alongside enhanced visual quality and improved security compared to previous standalone approaches. In this paper, we aim to address the following key questions:

1. How can the embedding process in image steganography be made more secure and adaptive to image content?
2. To what extent can the use of entropy and noise-based fused maps improve the imperceptibility of the stego image?
3. Can PSO effectively enhance the selection of embedding positions to improve PSNR and reduce BER?
4. Does the integration of Blowfish encryption with optimized embedding preserve the confidentiality and recoverability of the secret image?

The proposed steganographic system provides useful advantages to a range of operational contexts, including the industrial sector, public/government entities, and privately held companies, through its ability to transmit data securely while remaining undetectable. The system allows organizations to protect sensitive information, including medical records, financial data, and confidential communications through secured transmission that remains imperceptible to outside actors. The proposed system delivers real-time performance and robustness through a combination of lightweight encryption with optimization-driven adaptive embedding. These traits also make it ideal for deployment in secure image sharing, telemedicine, and digital forensics applications. The research enhances information security and privacy protection measures at a time when cyber threats are on the rise^16^.

Following this introduction, the paper is organized as follows. The second section covers existing research related to this work. The third section describes the methodology. Next, we present and analyze the results obtained through this method. Potential areas for future study are then outlined, while the sixth section concludes the paper.

## Related work

Recent studies on steganography have been focused on enhancing the practice through the integration of traditional LSB methods with contemporary encryption algorithms to boost security and robustness. In this context, Alanzy et al.^13^ proposed a multi-level steganography model that combines Advanced Encryption Standard (AES) and Blowfish encryption to protect secret messages before embedding them using LSB. Their approach leveraged a key-image mechanism and pixel-level randomization to increase resistance against visual and statistical steganalysis. The hybrid encryption scheme not only ensures the confidentiality of hidden data but also improves the quality of the stego image, as demonstrated by high PSNR values exceeding 85 dB and low MSE across different datasets. Furthermore, the method achieved efficient runtime performance compared to classical LSB and other benchmark techniques, proving its suitability for secure image communication scenarios.

The increasing complexity of Internet of Vehicles (IoV) communications requires immediate solutions to protect data confidentiality and ensure secure transmission. Traditional encryption methods demonstrate insufficient protection against new and emerging security threats. The research team of Rathore et al.^10^ developed Enhanced Automated Security Testing (EAST) as an efficient algorithm for secure transmission, which combines symmetric key encryption with steganography to protect real-time IoV data. The EAST model uses a custom block cipher to encrypt plaintext before embedding the resulting ciphertext into text-based stego files through Microsoft Word document color-based LSB manipulation. The proposed model outperformed AES, Data Encryption Standard (DES), Generalized Data Encryption Standard (G-DES), and standard LSB methods through its superior results in PSNR (up to 78.58%), time efficiency (0.086 ms), and avalanche effect (58.81%) while maintaining a reduced cover file size. The research demonstrated how encryption and steganography work together in a multi-layered security system and presented a hybrid solution that balances computational speed with steganalysis resistance. Their methodology also emphasized trust-based modeling, which is particularly relevant for vehicular networks with dynamic and decentralized structures.

Aparna and Madhumitha^8^ developed a security framework named Computational Intelligence for Enhanced Security Techniques (CIEST), which unites image encryption with steganography through multiple chaotic maps and DNA-based cryptography. The encryption process consists of two stages, which begin with DNA coding rules and chaotic key generation for secret image encoding, followed by confusion and diffusion achieved through combined chaotic maps. The encrypted image undergoes a chaos-guided LSB embedding process to hide itself inside a color cover image. The authors enhanced imperceptibility through two methods: they used multiple chaotic keys to permute different color channels of the cover before embedding and they randomized the selection of embedding positions. The authors demonstrated high resistance to statistical and differential attacks through metrics, such as entropy $\approx 7.9975$, PSNR (up to 56.1 dB), and SSIM $\approx 0.9992$. The authors demonstrated that CIEST surpasses traditional methods through exceptional randomness, robustness, and massive key space, which protects against brute-force attacks. The researchers demonstrated that image-based communication systems require chaos theory and DNA operations to achieve multi-layered security.

Islam et al.^6^ developed a hybrid steganographic model that merges traditional LSB embedding with visual cryptography to boost data confidentiality. The method is implemented differently than traditional LSB methods as the secret image is first transformed into an unreadable form (Share2) through an XOR operation with a randomly produced image (Share1). The LSB embedding process includes Share2, while Share1 functions as the cryptographic key needed to restore the original (secret) image. The two-share visual encryption system provides enhanced security because Share2 extraction by itself yields no useful information. The system enables both image and text concealment through Quick Response (QR) code conversion of messages before embedding. The evaluation results show excellent imperceptibility as PSNR values exceed 59 dB and SSIM scores reach 0.98 while maintaining extraction resistance when Share1 is absent. The authors demonstrated how visual cryptography and steganography can be employed together to provide multiple layers of security and privacy protection in image communication.

Younus and Hussain^4^ developed a hybrid image steganography system that combines cryptographic compression with an advanced embedding technique with security features such as an advanced embedding technique and Payload Capacity (PC) to ensure image imperceptibility. The preprocessing phase of this method consists of two stages, where the Vigenère cipher encrypts the secret message before Huffman coding compresses it to minimize message size and enhance payload efficiency. The scheme selects blocks and groups randomly through the knight tour algorithm and an arbitrary function to address traditional Exploiting Modification Direction (EMD) weaknesses. The actual embedding process employs an enhanced EMD method to embed encoded digits into the cover image by making minimal changes to pixel values. The evaluation results show this method outperformed traditional EMD, Optimized Exploiting Modification Direction (Opt EMD), and LSB-based schemes by achieving PSNR values above 55 dB, SSIM scores of approximately 0.99, and PC reaching 52,400 bytes without degrading image quality. The stego-images demonstrated near-identical statistical distributions to their original counterparts, according to chi-square steganalysis tests.

Subramanian et al.^2^ conducted a comprehensive analysis of advancements in image steganography by dividing methods into traditional, CNN-based, and Generative Adversarial Network (GAN)-based approaches. Traditional methods, such as LSB Pixel-Value Differencing (PVD), remain basic and provide only weak protection against attacks. The authors demonstrated that CNN-based models (e.g., U-Net and Xu-Net) and GAN-based frameworks (e.g., CycleGAN and SteganoGAN) achieved superior imperceptibility and steganalysis resistance as demonstrated by their reported PSNR values, which reached up to 64.7 dB. The authors stressed the importance of developing hybrid models and using advanced metrics and datasets to create more secure and efficient steganographic systems.

Zhang et al.^17^ developed a new adaptive steganographic algorithm to address the weaknesses that result from lossy image processing in Online Social Networks (OSNs), including JPEG recompression and enhancement filtering. The Minimizing Channel Error Rate (MINICER) technique separates channel errors into two distinct categories: steganography-related and steganography-independent. Their proposed method differs from previous methods as it embeds data into channel-processed covers and then replaces the corresponding original cover components to create the stego. The method preserves robustness while maintaining undetectability. The authors conducted extensive experiments using real-world platforms to show that their method outperformed Generalized Minimum-Error Adaptive Steganography (GMAS) and Joint Channel Rate and Image Scaling Steganography (JCRIS) in terms of message recovery accuracy and steganalysis resistance.

The trade-off between embedding capacity and imperceptibility in image steganography has been addressed in recent work by^18^ that combines cross-diagonal embedding PVD with the Modulus Function (MF) technique, guided by edge area patterns. This method aims to simultaneously enhance embedding efficiency and visual quality without compromising security. This approach was evaluated on 14 public datasets and achieved an average embedding capacity of 3.18 BPP, while maintaining high imperceptibility, with PSNR value exceeding 40 dB and SSIM scores above 0.98. The use of edge-aware block pattern embedding also contributed significantly to preserving visual fidelity. In addition to improved performance metrics, the method demonstrated robustness against RS steganalysis attacks, highlighting its strength in maintaining security under statistical detection.

The evaluation of classical steganographic techniques is useful for determining how embedding capacity interacts with imperceptibility and robustness. For example, Lu et al.^19^ performed a detailed evaluation of three spatial domain methods, including LSB, Most Significant Bit (MSB), and PVD across grayscale and color images. The authors evaluated the methods through PSNR, MSE, and SSIM tests using benchmark cover images including Lena, Baboon, and Peppers. The LSB technique produced the highest PSNR and SSIM values, which indicates superior visual quality and minimal distortion, while the PVD method provides higher embedding capacity. The MSB technique provides better data security than LSB but results in more distortion. This paper established a fundamental baseline for researchers to identify the effectiveness and limitations of common techniques that can support future work on robustness enhancement, computational efficiency improvement, and hybrid embedding approaches.

Other current research in robust steganography emphasizes error reduction in lossy environments, such as that of OSNs. MINICER, a new framework developed by Zeng et al.^20^, addresses both steganography-related and independent distortion sources by embedding messages in channel-processed covers and strategically replacing elements in the original image to maintain security and robustness. The method removes non-steganographic distortions (e.g., JPEG recompression and enhancement filtering) and uses the wet paper coding model to protect unstable elements from modification. The experimental results showed better performance in terms of message recovery and detection resistance than JCRIS and GMAS in real-world platforms. MINICER achieved robustness through its design, which eliminates the requirement for error correction codes while preserving high imperceptibility and low error rates during aggressive image post-processing.

Yuan et al.^21^ developed Attack and Deep Fusion for Image Steganography (ADF-IS), which combines a Universal Adversarial Network (UAN) with pixel-wise deep fusion to create a GAN-based image-steganography system that defends against CNN steganalyzers while maintaining high PC and imperceptibility. The system consists of four modules that include the attack (UAN) for producing universal adversarial perturbations to confuse CNN steganalyzers and the Encoder (generator) for developing pixel-wise fusion ”stego masks” that focus on edge and texture areas, the Critic (discriminator), which uses Wasserstein GAN training to provide stable adversarial feedback, and the Decoder for message reconstruction. The system uses a combined loss function that combines cover–stego distortion (MSE) with generator loss and recovery accuracy (cross-entropy) through specific weight values ($\alpha$=0.2, $\beta$=1, $\gamma$=0.04).

Chahar et al.^22^ presented an explainable DL system for image steganography that optimizes all four essential factors of imperceptibility, robustness, interpretability, and usability. The system combines a modified CycleGAN for reversible embedding and extraction with attention modules that generate human-readable heatmaps of embedded areas and implements a multi-objective loss function that unites adversarial indistinguishability with cycle consistency and image-fidelity constraints, and usability constraints that restrict computational overhead. The system is trained through simulated bit streams on BOSSbase 1.01 data. Before implementation, the developers used PyTorch for performance evaluation through PSNR and SSIM metrics, including steganalysis accuracy assessment, user study assessment of task duration, usability, satisfaction, and interpretability. The method produces PSNR values of approximately 41.2 dB and SSIM values of approximately 0.984, outperforming LSB and Discrete Cosine Transform (DCT) methods while showing 57.8% resistance to contemporary steganalyzers. The attention visualizations obtained high user ratings at 4.6 out of 5 points, while the system demonstrated practical usability through 18.2 seconds of average task duration. Users rated ease-of-use at 4.5 out of 5 and satisfaction at 4.6 out of 5. The research established explainability and usability as fundamental design priorities for learned steganography systems, but acknowledges the study’s restricted dataset and small participant group while recommending future work on multiple media types and expanded research participant numbers.

Research into DL methods, particularly GANs for enhancing image steganography security and adaptability, has expanded significantly over the past few years. The embedding algorithms S-UNIWARD, HILL, and MiPOD use fixed cost functions based on local image structures, which restrict their adaptability and resistance to steganalysis attacks. Wang et al.^23^ developed a GAN-based framework that teaches adaptive embedding cost maps through a dual-stream U-Net generator that combines Convolutional Spatial Attention (CSA) with image enhancement modules. The research builds upon the Automatic Steganographic Distortion Learning framework with GAN (ASDL-GAN) and UT-GAN models, which enhanced cost learning through adversarial training and architectural improvements. Yet, it faced challenges with training stability and detection precision. The research of Steganographic Pixel-wise Actions and Rewards with Reinforcement Learning (SPAR-RL) and CF-UT-GAN explored reinforcement learning and feedback-based optimization to enhance embedding decisions. It focused on detailed attention to edge and texture regions, leading to better cost map accuracy. The experimental findings showed that their approach boosts resistance to sophisticated steganalysis models, thus creating a major breakthrough in adaptive image steganography with attention guidance.

## Methodology

**Fig. 2:**
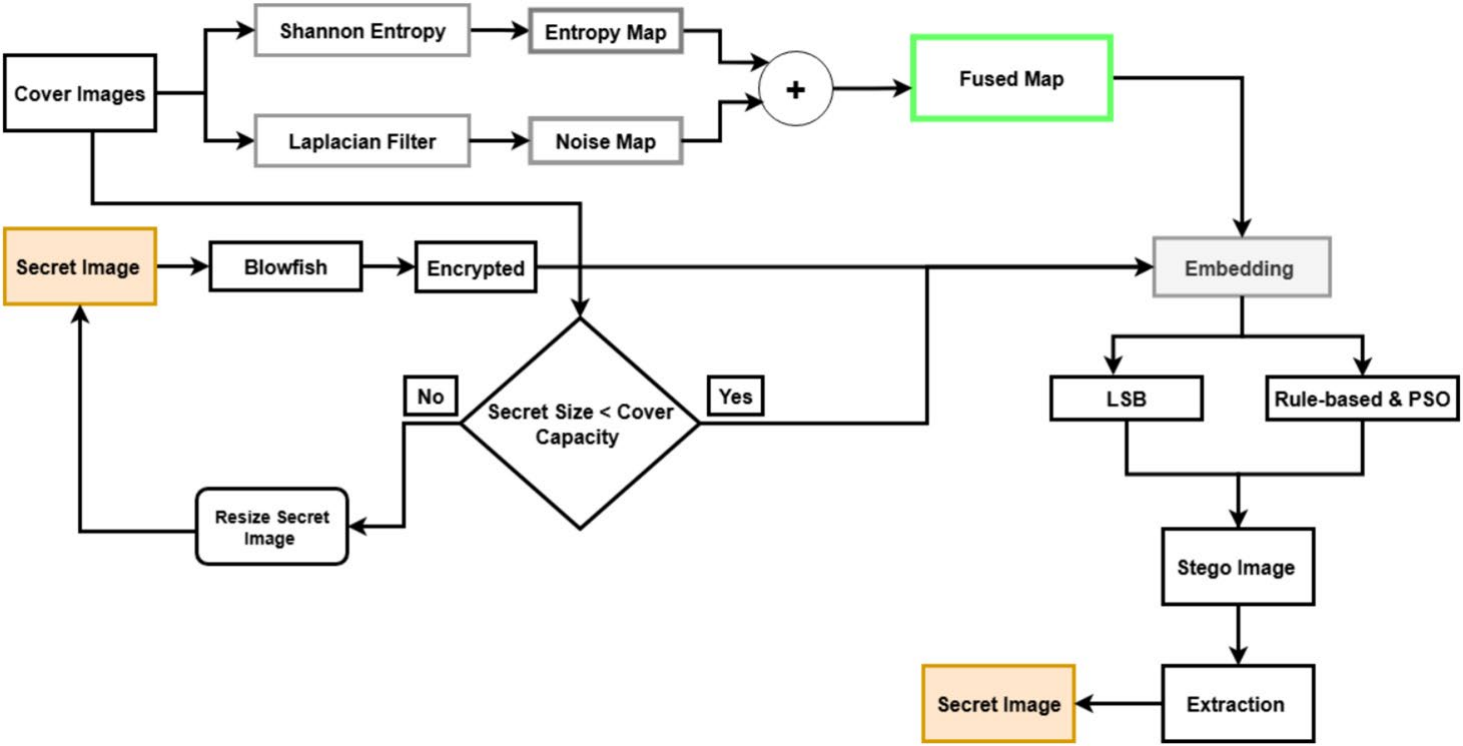
Proposed approach.

The proposed methodology as shown in Fig. 2 consists of different steps: data set preparation and preprocessing, fused map generation on the cover images, secret image encryption, secret image embedding within cover image using adaptive LSB embedding, optimization embedding with PSO, extraction of the secret image from the cover image, and finally, the evaluation process. In our work, the Blowfish cipher serves as a symmetric block encryption algorithm to encrypt the secret image as it maintains confidentiality when hidden data extraction occurs. The encrypted data is converted into a binary bitstream for embedding purposes. In the embedding process, we embed the encrypted secret image within the cover image based on the fused map guidance, which combines two cover image feature maps: an entropy map for local complexity detection and a Laplacian-based noise map for edge region identification. The data embedding process takes place in areas that are both perceptually less sensitive and information-rich through the fusion technique. The next step involves PSO to find the most suitable sequence of pixel indices for embedding secret bits. The swarm contains particles that function as different index permutation candidates for the attention-prioritized elements. The fitness function assesses each solution through PSNR and BER evaluation to maximize both visual quality and extraction accuracy. The extracted solution from PSO serves as the basis for embedding the bitstream into the LSBs of the cover image. The extraction process starts by extracting embedded bits from their optimized positions before converting them back into bytes. The Blowfish key serves to decrypt the encrypted content, which reveals the original secret image. The system assessment includes multiple objective metrics that evaluate its imperceptibility through PSNR, SSIM, MSE, BER, IF, and execution time to measure its robustness and overall performance.

### Dataset preparation and acquisition

We conduct testing operations on two different datasets throughout this work. The USC-SIPI image database, which contains digitized images, serves as the secret images dataset^23,24^. The database contains volumes that organize images according to their nature. The images in each volume have different pixel dimensions, which include 256$\times$256 pixels, 512$\times$512 pixels, and 1024$\times$1024 pixels. The USC-SIPI Volume 3 miscellaneous page allows users to download this dataset through ( https://sipi.usc.edu/database/database.php? volume=misc)).

The BOSSbase dataset contains 10,000 grayscale images that measure 512$\times$512 pixels and exist in Portable Gray Map (PGM) format^25^. Seven different cameras took the images before the conversion to JPG format. The BOSSbase dataset [Version 1.01] is the most current version available. The website https://www.kaggle.com/datasets/lijiyu/BossBase allows users to download this image. The chosen datasets, BOSSbase and USC-SIPI, were selected as they represent a broad spectrum of image characteristics that are important for steganography. BOSSbase provides high-resolution natural grayscale images, which helps evaluate real-world imperceptibility. The USC-SIPI database provides synthetic and structured images with controlled edge and texture properties, which makes it appropriate for evaluating edge-based embedding strategies. The proposed method demonstrates robustness and adaptability to different image types and conditions because of the diversity of the dataset.

### Preprocessing and encryption

The system accepts both a cover image and a secret image during its preprocessing and encryption operation. First, it adjusts the secret image dimensions to match the cover image embedding capacity. The Blowfish symmetric encryption algorithm encrypts the resized secret image using a shared secret key to protect the data from unauthorized access. The encryption process produces ciphertext, which gets converted into a binary bitstream before the embedding operation in the following step. Algorithm 1 shows that the Blowfish encryption process starts by setting P-array and S-boxes with predefined constants before key expansion to customize these structures. The secret key expansion process follows the initialization of fixed constants for the P-array and S-boxes. The plaintext undergoes 16 Feistel rounds of processing through two 32-bit halves, which apply XOR operations and the non-linear function F using the S-boxes. The final swap operation and two additional XOR steps generate the ciphertext after completing all rounds. The design of this structure enables efficient encryption and decryption operations, which makes Blowfish appropriate for secure data embedding applications. The Blowfish encryption module in our implementation used a fixed 128-bit (16-byte) key size for encryption. A different 128-bit key was produced through a secure pseudo-random number generator for each embedding test. The 128-bit key configuration of Blowfish was selected for our experiments as it provides both high security and efficient processing while maintaining a uniform testing environment^26^. Algorithm 2 shows the Blowfish decryption process.

**Algorithm 1:**
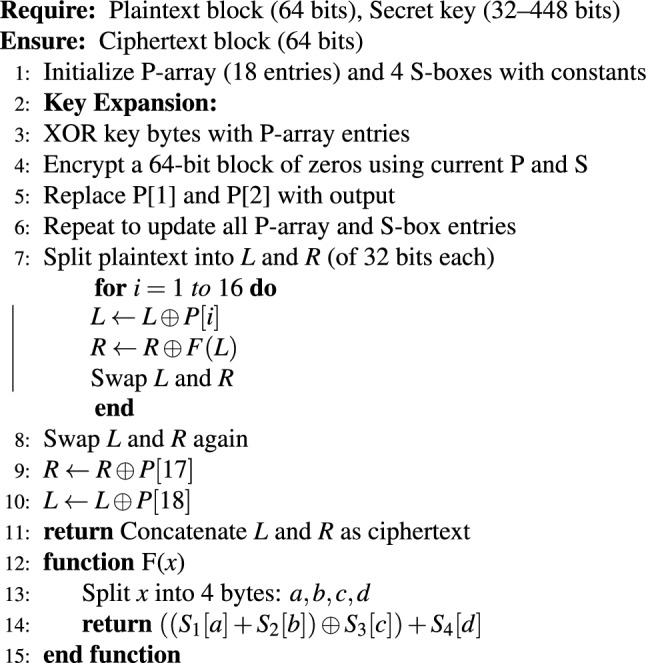
Blowfish encryption (Pseudocode).

**Algorithm 2:**
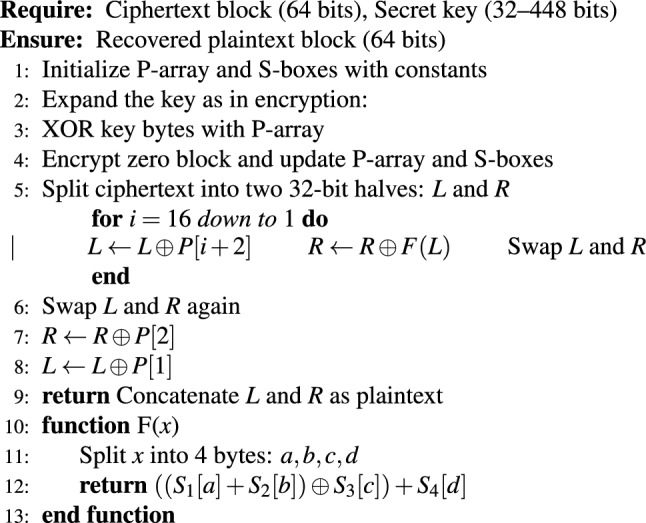
Blowfish decryption (Pseudocode).

### Fused map generation using entropy and noise maps

The fused map (entropy and noise combined) generation stage produces two feature maps from the cover image, which serve as guidance for embedding operations. The entropy map serves as the first feature, which measures pixel region texture complexity through Shannon entropy calculations. The noise map emerges from Laplacian operator processing, which strengthens both edge intensity and high-frequency details. The two maps receive normalization to match scales before they combine through weighted fusion to produce a single fused map. The combined map identifies the most important image areas, which are least noticeable to human perception, thus making them suitable for embedding secret data.

The proposed system produces a fused map through uniting entropy-based and noise-based feature maps of the cover image to locate areas that are less sensitive to perception and contain abundant texture for embedding. The entropy map *E*(*i*, *j*) is calculated over a local window centered at pixel (*i*, *j*) using Shannon’s entropy:

1 $$\begin{aligned} E(i,j) = - \sum _{k=0}^{255} p_{k}(i,j) \cdot \log _{2}\big (p_{k}(i,j)\big ), \end{aligned}$$

where $p_{k}(i,j)$ denotes the probability of gray level *k* occurring within the neighborhood window around pixel (*i*, *j*).

The noise map *N*(*i*, *j*) is obtained by applying the Laplacian operator to capture high-frequency variations such as edges and fine details:

2 $$\begin{aligned} N(i,j) = \left| \nabla ^{2} I(i,j) \right| , \end{aligned}$$

where *I*(*i*, *j*) is the intensity value at pixel (*i*, *j*) and $\nabla ^{2}$ is the Laplacian operator. Both maps are normalized into the range [0, 1] to ensure scale compatibility.

The fused map *F*(*i*, *j*) is then obtained by a weighted linear combination:

3 $$\begin{aligned} F(i,j) = \alpha \cdot \hat{E}(i,j) + (1-\alpha ) \cdot \hat{N}(i,j), \end{aligned}$$

where $\hat{E}(i,j)$ and $\hat{N}(i,j)$ are the normalized entropy and noise maps, respectively, and $\alpha \in [0,1]$ is a tunable parameter controlling the relative importance of entropy versus noise features. The fusion approach directs embedding operations toward areas that present both complex textures and structural changes to enhance both imperceptibility and robustness.

### Data embedding and extraction

Our novel system implements a content-dependent priority-based LSB substitution method, which embeds 1 bit per chosen pixel. The system generates two guidance cues from the cover image through local entropy analysis of 9$\times$9 windows and Laplacian-based noise detection and edge response evaluation before combining them through a weighted convex operation with parameter $\alpha$. The normalized fused map undergoes an exponentiation operation with parameter $\gamma$ before the pixels receive descending order ranking to generate their priority values. The secret image undergoes Blowfish encryption to produce the payload from image content before the resulting bitstream gets embedded into the LSB positions of priority-ranked pixels until the payload reaches its end.

The extraction process reads LSB values from pixels in their original priority sequence to maintain synchronization. The system uses fixed 1-bit capacity per pixel while adapting through spatial selection of pixels. The optimization process uses PSO to find the best values for parameters $\alpha$ and $\gamma$ within their ranges [0,1] and [0.5,5] to minimize visual distortion. The PSO system optimizes the map through $\alpha$ and $\gamma$ parameters to direct both embedding and extraction operations using priority indices, which results in better PSNR/SSIM performance at equivalent effective bits per pixel. The extraction process retrieves embedded bits from their original optimized positions to maintain synchronization between the sender and receiver. The recovered bitstream gets reconstructed into bytes through decryption using the shared Blowfish key to achieve high accuracy in restoring the original secret image.

### Optimization embedding using PSO

In the embedding optimization stage, we transform the fused attention map into priority indices that show the most suitable pixels for data embedding through flattening and sorting operations. PSO is used to optimize the selection process by trying different index permutations to find the best embedding sequence. The swarm contains candidate solutions, which represent different pixel position orders, and their quality is determined by a fitness function that evaluates both PSNR for visual quality and BER for extraction accuracy. The PSO algorithm finds the optimal solution through repeated updates, which results in an embedding order that maintains both imperceptibility and robustness^27,28^.

The PSO Algorithm 3 draws inspiration from the social behavior and movements of birds and fish. It uses candidate solutions known as particles to explore the search space through velocity and position updates that combine individual experiences with swarm-wide knowledge. The position of each particle changes based on its personal best position and the global best position discovered throughout the process. The velocity update process depends on three elements, including inertia (previous velocity), cognition (personal experience), and learned social behavior (swarm intelligence). The iterative updates of particles lead them to find the optimal solution. The algorithm has gained popularity because it offers simplicity and efficiency while handling complex nonlinear optimization problems with minimal parameter adjustments.

The PSO algorithm parameters for embedding optimization are presented in Table 1. The inertia weight value is used to achieve an optimal balance between exploration and exploitation during the search process. The inertia weight was set to $w=0.72$ in order to provide a suitable balance between exploration and exploitation during the search process. The acceleration coefficients were configured as $c_{1}=1.49$ (cognitive) and $c_{2}=1.49$ (social), following the constriction factor approach, which has been shown to ensure stable convergence behavior. The swarm size remains constant at N = 18 particles to achieve a balance between search diversity and computational efficiency, while the maximum number of iterations was set to 25 for fast convergence. The selected parameter settings remained constant throughout all experiments to maintain result reproducibility and enable comparison between results.

**Table 1:** PSO parameter settings used in embedding optimization.

Parameter	Value
Inertia weight (*w*)	0.72
Cognitive coefficient ($c_{1}$)	1.49
Social coefficient ($c_{2}$)	1.49
Number of particles (*N*)	18
Maximum iterations	25

In the proposed optimization process, as shown in Algorithm 3, the PSO algorithm evaluates each candidate solution (defined by the fusion weight $\alpha$ and the exponent $\gamma$) through a fitness function that measures the visual distortion introduced by embedding. Specifically, the fused map *F*(*i*, *j*) that is generated from the entropy and noise features is transformed into priority indices using the parameter $\gamma$. These indices are then used to embed the encrypted bitstream into the cover image, producing a stego image. The fitness, as shown in Equation 4, of each particle is computed as the MSE between the original cover image *I* and the corresponding stego image *S*:

4 $$\begin{aligned} \text {Fitness}(\alpha ,\gamma ) = \text {MSE}(I,S) = \frac{1}{mn} \sum _{i=1}^{m} \sum _{j=1}^{n} \big (I(i,j) - S(i,j)\big )^{2}. \end{aligned}$$

The fitness of the algorithm improves when MSE values decrease because this leads to better imperceptibility. The optimization goal directly targets distortion reduction for embedding while maintaining high PSNR and SSIM values, which ensures robustness and imperceptibility in the embedding process.

**Algorithm 3:**
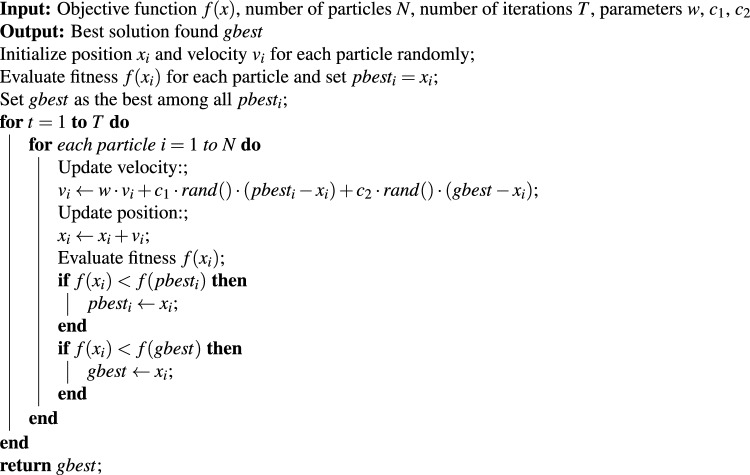
Particle swarm optimization (PSO) algorithm.

The optimization process of embedding uses PSO to find the best combination of fusion and priority parameters. The PSO parameters follow the recommendations from the existing scientific literature to achieve stable convergence and maintain proper exploration-exploitation behavior. The inertia weight $w=0.72$ and the acceleration coefficients $c_{1}=1.49$ (cognitive) and $c_{2}=1.49$ are selected from the constriction factor method proposed by Clerc and Kennedy^29^, as it delivers optimal results for local and global search operations. The selected parameter values produce stable convergence and prevent early termination during validation image tests. The PSO parameters remain constant throughout all experiments because this approach ensures both result reproducibility and comparison consistency.

**Fig. 3:**
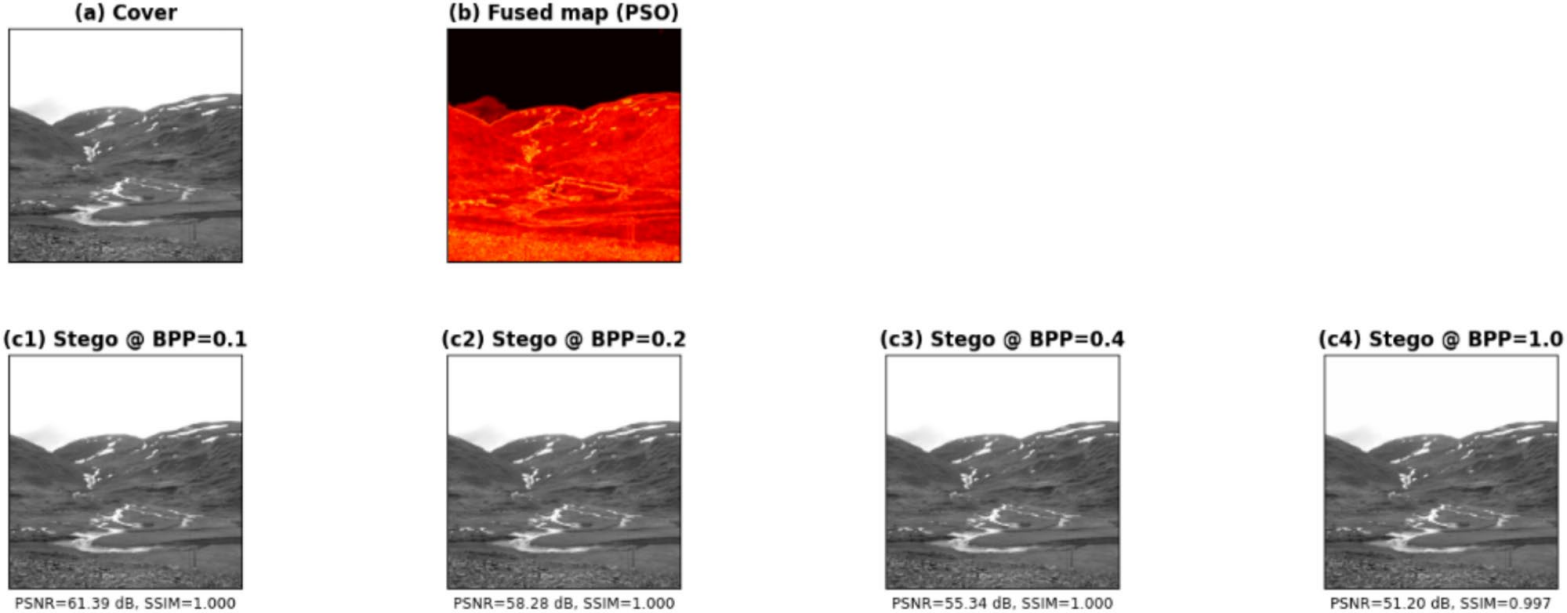
Cover image and stego image at different BPP targets. Images were obtained from the open-source BOSSBase dataset and used for academic research purposes.

Figure 3 demonstrates the proposed steganography method that uses fused-map guidance. The grayscale cover image is shown in Panel (a). The PSO-generated fused map in Panel (b) indicates high embedding priority through hot colors while maintaining dark areas to preserve visual quality. The stego images in Panels (c1–c4) demonstrate the effect of increasing embedding rates (BPP) from lowest to highest. The fused map directs modifications to rich areas while smooth sections of the image remain unchanged. The arrangement demonstrates how the guidance map acts as an adaptive mask, which distributes the payload based on image structure to maintain imperceptibility at different capacity levels.

**Fig. 4:**
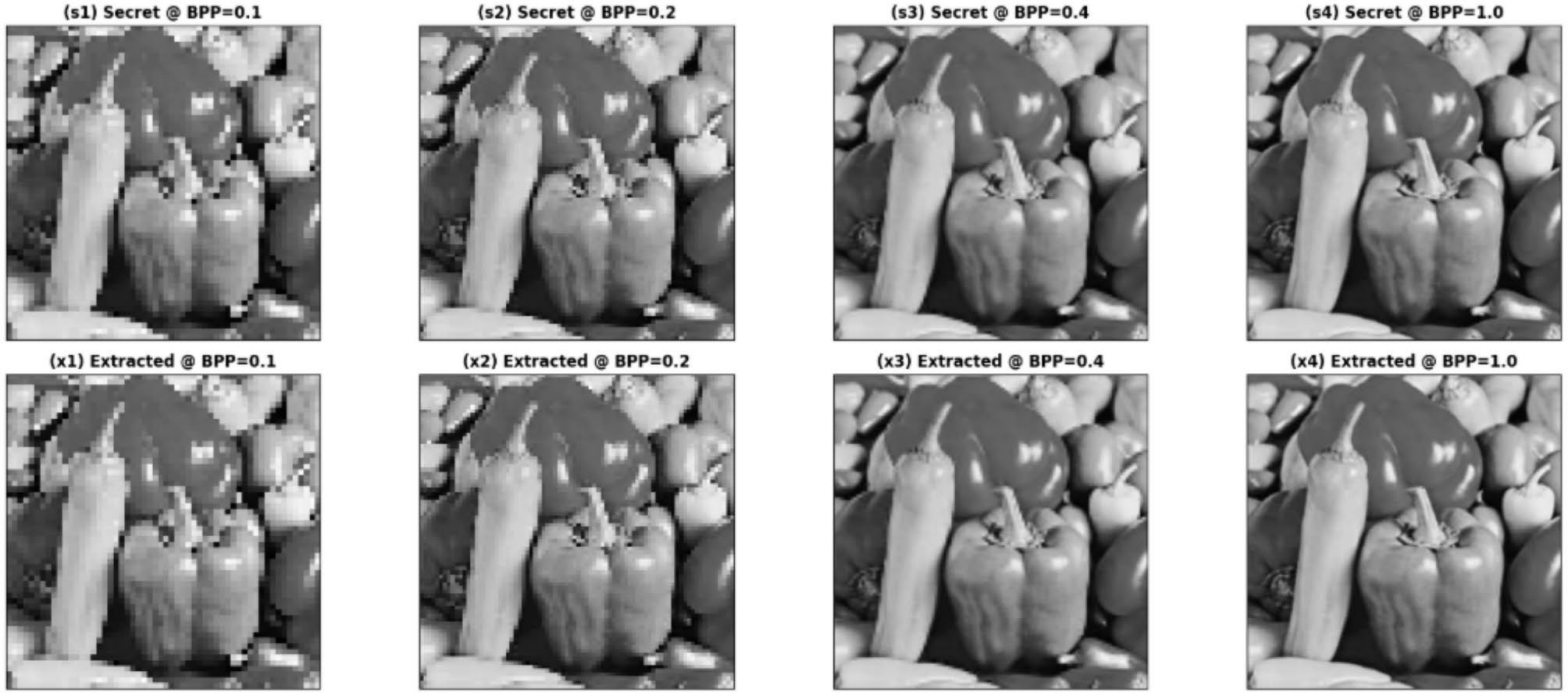
Secret image and extracted secret image at different BPP target. Images were obtained from the open-source USC-SIPI dataset and used for academic research purposes.

On the other hand, Fig. 4 demonstrates a qualitative assessment of secret-image recovery at four different embedding rates (BPP). The top section (s1–s4) displays the secret images before embedding while the bottom section (x1–x4) shows the images recovered from the stego images using the decoder. The extracted results maintain the spatial arrangement, edge details, and object boundaries of their original secret images throughout all payload levels, which indicates successful recovery of essential content. The extraction pipeline shows high perceptual agreement throughout all payload levels because the increased embedding load produces only minor visual artifacts that include slight smoothing and local contrast variations.

## Results

The evaluation of the proposed method used four assessment criteria, including imperceptibility, capacity, security, and robustness. The evaluation of imperceptibility used four metrics (i.e., MSE, PSNR, SSIM, and IF). The evaluation of capacity depended on two metrics, PC and BPP. The security evaluation depended on steganalysis methods, including RS analysis and two DL detectors named Xu-Net and Ye-Net. The following section explains the evaluation of robustness through standard distortion tests, as well as presents all the performance metrics by testing different secret image sizes and different BPP at 0.1, 0.2, 0.4, and 1.0.

The current implementation works with grayscale images through priority-guided 1-bit-per-pixel LSB embedding that uses a PSO-optimized fused map (entropy + Laplacian) with parameters $(\alpha , \gamma )$. The same framework can be used for color image embedding through two methods: (a) YCbCr luminance-chrominance space embedding in chroma channels, because color changes are less noticeable to humans, or (b) Red Green Blue (RGB) channel-specific fused maps with individual budgets for each color channel. The optimization process for PSO determines channel-specific parameters $(\alpha _c, \gamma _c)$ for each color channel Y, Cb, Cr (or R, G, B) while maintaining color consistency through channel modification limits and balanced pixel transformations.

The selection-based guidance remains unchanged for compressed delivery, but the actual embedding process shifts to transform domains (DCT/ Discrete Wavelet Transform (DWT)) to enhance robustness, while PSO optimizes selection weights and exponents for transform-domain activity measures. The method remains computationally manageable when dealing with increasing numbers of channels and frames through vectorization and GPU acceleration of entropy or noise calculations and fitness evaluations, and PSO starts from the previous image/frames and focuses on the top K locations per tile. The method maintained its fundamental operation through PSO-guided parameter optimization of $(\alpha , \gamma )$ and priority-based embedding while achieving practical use for color content.

### Imperceptibility

**MSE:** is used to represent the average of the MSE between the pixels of the cover image and the stego image. Here, the MSE value is calculated by using Equation 5. The range of MSE value is between 0 and 1, with the lower values (those closer to 0) are better than the higher values (those closer to 1). The MSE utilized in our calculations directly used grayscale pixel intensities that exist within the standard 8-bit range from 0 to 255. The calculation of MSE did not require any normalization step to transform the values into the range of 0 to 1. The selection maintains alignment with PSNR and other full-reference image quality metrics because they operate under the assumption that pixel values exist between 0 and 255.

^2,4^. MSE can be calculated as follows:

5 $$\begin{aligned} \text {MSE} = \frac{1}{mn} \sum _{i=1}^{m} \sum _{j=1}^{n} (I(i,j) - K(i,j))^2 \end{aligned}$$

where:

$$\begin{aligned}&\text {MSE}: \text {Mean Squared Error} \\&m, n: \text {Image dimensions} \\&I(i,j): \text {Pixel value of the original image} \\&K(i,j): \text {Pixel value of the stego image} \end{aligned}$$

**PSNR:** is used to describe the quality of the stego-image based on the Human Visual System (HVS), where its normal value is 30 dB^10,13^. When the PSNR value is greater than 30 dB, this indicates the data inserted in the image is invisible to the human eye^15,30^. The PSNR value can be calculated by Equation 6; the higher values of PSNR are better than the lower values. PSNR can be calculated as follows^24,31^:

6 $$\begin{aligned} \text {PSNR} = 10 \cdot \log _{10} \left( \frac{{\text {MAX}}^2}{{\text {MSE}}} \right) \end{aligned}$$

where:

$$\begin{aligned}&\text {PSNR}: \text {Peak Signal-to-Noise Ratio} \\&\text {MAX}: \text {Maximum possible pixel value} \\&\qquad \qquad \text {Mean Squared Error between original and}\\&\text {MSE}: \qquad \qquad \qquad \text {stego images} \end{aligned}$$

**SSIM:** is used to measure the similarity between the cover image and the stego image. The result of the SSIM value remains between 0 and 1. When the SSIM value is close to 1, it means that the stego image is similar to the cover image and is of high quality. The SSIM value is calculated using Equation 7^25,32^.

7 $$\begin{aligned} \text {SSIM}(I, K) = \frac{{(2\mu _I\mu _K + C_1)(2\sigma _{IK} + C_2)}}{{(\mu _I^2 + \mu _K^2 + C_1)(\sigma _I^2 + \sigma _K^2 + C_2)}} \end{aligned}$$

where:

$$\begin{aligned}&I, K: \text {Two different images} \\&\text {SSIM}(I, K): \text {Structural Similarity Index Measure} \\&\mu _I, \mu _K: \text {Mean intensity of images } I \text { and } K \\&\qquad \qquad \text {Standard deviation of intensities of images}\\&\sigma _I, \sigma _K: \qquad \qquad \qquad \quad {I \text { and } K} \\&\sigma _{IK}: \text {Covariance of intensities of images } I \text { and } K \\&C_1, C_2: \text {Constants to stabilize the division} \end{aligned}$$

**IF:** is another metric to identify the quantity of errors in pixel values. If the value is closer to 1, it means that the two images are more similar. The IF value is calculated using Equation 8^33,34^.

8 $$\begin{aligned} \text {IF}(I, K) = \frac{1}{N} \sum _{i=1}^{N} \left( 1 - \frac{|I_i - K_i|}{\max (I_i, K_i)}\right) \end{aligned}$$

where:

$$\begin{aligned}&\text {IF}(I, K): \text {Information Fidelity between images } I \text { and } K \\&N: \text {Total number of pixels in the images} \\&I_i, K_i: \text {Pixel values at position } i \text { in images } I \text { and } K \end{aligned}$$

### Capacity

**PC:** is a measure of how much data can be hidden in the cover image by the proposed steganography; this will be very dependent on the size of the cover image. Equation 9 shows the calculation of this measure^35,36^.

9 $$\begin{aligned} \text {PC} = {{\text {T} \times N \times 8}} \end{aligned}$$

where:

$$\begin{aligned}&\text {T}: \text {Total pixels of cover image} \\&\qquad \text {bytes per pixels: 1 for grayscale or 3 for color }\\&N: \qquad \qquad \qquad \qquad { \text { image }} \\ \end{aligned}$$

**BPP:** is the average value of the embedding per pixel of the whole image^37,38^. BPP can be calculated by Equation 10^4^.

10 $$\begin{aligned} \text {BPP} = \frac{{\text {Payload Capacity (bits)}}}{N} \end{aligned}$$

where:

$$\begin{aligned}&\text {BPP}: \text {Bits Per Pixel} \\&\text {Payload Capacity (bits)}: \text {Total payload capacity in bits} \\&N: \text {Total number of pixels in the image} \end{aligned}$$

Table 2 summarizes performance across payloads without PSO and shows the expected trade-off between embedding rate and cover quality: as $\textrm{BPP}$ increases from $\approx 0.10$ to $\approx 1.0$, $\textrm{PSNR}_{\textrm{cs}}$ decreases by roughly $10\,\textrm{dB}$ (from $\sim 61.3\,\textrm{dB}$ to $\sim 51.2\,\textrm{dB}$) and $\textrm{MSE}_{\textrm{cs}}$ rises ($\approx 0.048 \rightarrow 0.49$), while perceptual similarity remains essentially intact with $\textrm{SSIM}_{\textrm{cs}}\ge 0.997$ and $\textrm{IF}_{\textrm{cs}}\approx 0.99998$ across all settings. Secret recovery is lossless in every case, evidenced by $\textrm{PSNR}_{\textrm{sec}}=\infty$ and $\textrm{SSIM}_{\textrm{sec}}=1.0000$. For a fixed payload, results are practically identical to the secret image’s spatial size ($64\times 64$, $128\times 128$, $512\times 512$), indicating that, after encryption, the distortion is governed by the bit budget rather than the content or dimensions of the secret image. The system shows expected behavior because payload size directly affects both embedding and extraction times through its impact on modified and read positions. The used payload amounts to 0.196, which matches the target value while staying below it because of byte/block alignment and host image capacity restrictions (Capacity = 262,144 bits). The system produces high-quality stego images through perfect secret recovery across various payload amounts, even without PSO implementation. The system requires spatial guidance or parameter optimization to minimize MSEcs at elevated BPP levels without compromising recoverability.

**Table 2:** Combined summary per BPP for cover–stego and secret–extracted metrics using different secret image sizes without PSO.

Secret Size	$\hbox {BPP}_{{tgt}}$	$\hbox {BPP}_{{used}}$	$\hbox {PSNR}_{{cs}}$ (dB)	$\hbox {SSIM}_{{cs}}$	$\hbox {MSE}_{{cs}}$	$\hbox {IF}_{{cs}}$	$\hbox {PSNR}_{{sec}}$	$\hbox {SSIM}_{{sec}}$	$t_{embed}$ (ms)	$t_{extract}$ (ms)	Capacity (bits)	Target (bits)	Used (bits)
64$\times$64	0.10	0.096	61.28	1.0000	0.0484	0.999998	$\infty$	1.0000	35.71	21.71	262,144	26,214	25,152
64$\times$64	0.20	0.196	58.22	0.9999	0.0979	0.999997	$\infty$	1.0000	27.76	18.16	262,144	52,429	51,264
64$\times$64	0.40	0.383	55.31	0.9996	0.1913	0.999994	$\infty$	1.0000	51.42	33.12	262,144	104,858	100,416
64$\times$64	1.00	0.989	51.21	0.9972	0.4918	0.999984	$\infty$	1.0000	134.82	90.51	262,144	262,144	259,264
128$\times$128	0.10	0.096	61.32	1.0000	0.0480	0.999998	$\infty$	1.0000	13.05	8.70	262,144	26,214	25,152
128$\times$128	0.20	0.196	58.23	0.9999	0.0978	0.999997	$\infty$	1.0000	26.74	17.13	262,144	52,429	51,264
128$\times$128	0.40	0.383	55.30	0.9996	0.1918	0.999994	$\infty$	1.0000	51.72	34.57	262,144	104,858	100,416
128$\times$128	1.00	0.989	51.19	0.9972	0.4941	0.999984	$\infty$	1.0000	134.60	95.23	262,144	262,144	259,264
512$\times$512	0.10	0.096	61.32	1.0000	0.0480	0.999998	$\infty$	1.0000	30.00	19.05	262,144	26,214	25,152
512$\times$512	0.20	0.196	58.26	0.9999	0.0971	0.999997	$\infty$	1.0000	58.88	41.81	262,144	52,429	51,264
512$\times$512	0.40	0.383	55.30	0.9996	0.1918	0.999994	$\infty$	1.0000	119.05	75.70	262,144	104,858	100,416
512$\times$512	1.00	0.989	51.19	0.9972	0.4947	0.999984	$\infty$	1.0000	302.60	195.98	262,144	262,144	259,264

$\hbox {PSNR}_{sec}$ shown as $\infty$ indicates lossless recovery of the secret (no measurable error).

Table 3 reports the results with PSO and confirms the expected payload-distortion trade-off while preserving excellent perceptual quality and perfect recoverability of the secret. As $\textrm{BPP}$ increases from $\approx 0.10$ to $\approx 1.0$, $\textrm{PSNR}_{\textrm{cs}}$ decreases from $\sim 61.4\,\textrm{dB}$ to $\sim 51.2\,\textrm{dB}$ and $\textrm{MSE}_{\textrm{cs}}$ rises ($\approx 0.047 \rightarrow 0.493$), yet $\textrm{SSIM}_{\textrm{cs}}\ge 0.997$ and $\textrm{IF}_{\textrm{cs}}\approx 0.99998$ across all settings, which indicates that cover distortion remains visually negligible. Secret reconstruction is lossless in every case ($\textrm{PSNR}_{\textrm{sec}}=\infty$, $\textrm{SSIM}_{\textrm{sec}}=1.0000$). The performance remains constant for fixed payloads as the encryption process produces observable distortions that depend on the bit budget instead of the secret content or dimensions (64$\times$64, 128$\times$128, 512$\times$512). The extraction and embedding operations require more time because of the increased number of positions that need modification or reading, yet their minimal variations stem from system implementation factors. The PSO method produces small performance improvements compared to the no-PSO configuration at low-to-mid payload levels, which results in 0.01–0.15 dB improvements in PSNRcs and 1–3% MSEcs reductions. The highest payload achieves parity between PSO and no-PSO methods because parameter optimization can eliminate remaining distortion without affecting data capacity or complete secret retrieval.

**Table 3:** Combined summary per BPP for cover–stego and secret–extracted metrics using different secret image sizes with PSO.

Secret Size	$\hbox {BPP}_{{tgt}}$	$\hbox {BPP}_{{used}}$	$\hbox {PSNR}_{{cs}}$ (dB)	$\hbox {SSIM}_{{cs}}$	$\hbox {MSE}_{{cs}}$	$\hbox {IF}_{{cs}}$	$\hbox {PSNR}_{{sec}}$	$\hbox {SSIM}_{{sec}}$	$t_{embed}$ (ms)	$t_{extract}$ (ms)	Capacity (bits)	Target (bits)	Used (bits)
64$\times$64	0.10	0.096	61.40	1.0000	0.0471	0.999999	$\infty$	1.0000	15.53	8.61	262,144	26,214	25,152
64$\times$64	0.20	0.196	58.29	0.9999	0.0964	0.999997	$\infty$	1.0000	50.36	18.17	262,144	52,429	51,264
64$\times$64	0.40	0.383	55.34	0.9996	0.1900	0.999994	$\infty$	1.0000	50.85	39.38	262,144	104,858	100,416
64$\times$64	1.00	0.989	51.21	0.9972	0.4923	0.999984	$\infty$	1.0000	135.89	113.97	262,144	262,144	259,264
128$\times$128	0.10	0.096	61.41	0.9999	0.0470	0.999999	$\infty$	1.0000	13.24	9.46	262,144	26,214	25,152
128$\times$128	0.20	0.196	58.30	0.9998	0.0962	0.999997	$\infty$	1.0000	26.37	18.38	262,144	52,429	51,264
128$\times$128	0.40	0.383	55.35	0.9996	0.1896	0.999994	$\infty$	1.0000	56.27	39.22	262,144	104,858	100,416
128$\times$128	1.00	0.989	51.21	0.9972	0.4916	0.999984	$\infty$	1.0000	136.14	108.37	262,144	262,144	259,264
512$\times$512	0.10	0.096	61.39	1.0000	0.0472	0.999998	$\infty$	1.0000	13.04	9.40	262,144	26,214	25,152
512$\times$512	0.20	0.196	58.28	0.9999	0.0966	0.999997	$\infty$	1.0000	26.85	18.09	262,144	52,429	51,264
512$\times$512	0.40	0.383	55.34	0.9995	0.1900	0.999994	$\infty$	1.0000	51.13	33.88	262,144	104,858	100,416
512$\times$512	1.00	0.989	51.20	0.9972	0.4928	0.999984	$\infty$	1.0000	136.14	108.37	262,144	262,144	259,264

$\hbox {PSNR}_{{sec}}$ shown as $\infty$ indicates lossless recovery of the secret (no measurable error).

Table 4 shows the proposed method maintains perfect secret reconstruction while achieving imperceptible cover image distortion through payloads ranging from 0.1 to 1.0 with a $64\times 64$ secret and $N=30$ test images. The proposed method maintains cover image quality at ($\textrm{PSNR}_{c\rightarrow s}$) decreases monotonically from $61.39\,\textrm{dB}$ to $51.21\,\textrm{dB}$ as $\textrm{BPP}$ increases, yet $\textrm{SSIM}_{c\rightarrow s}\approx 1.000$ and $\textrm{IF}_{c\rightarrow s}\approx 1.000$ at all rates, indicating negligible perceptual impact despite the expected rise in $\textrm{MSE}_{c\rightarrow s}$ ($\approx 0.047\rightarrow 0.492$). Secret recovery is lossless in every scenario, with $\textrm{SSIM}_{\textrm{sec}}=1.000$ and $\textrm{PSNR}_{\textrm{sec}}=\infty$, the latter reflecting $\textrm{MSE}_{\textrm{sec}}=0$ (the reported ”inf±nan” stems from undefined variance under perfect reconstruction). Reported dispersions are minuscule (e.g., $\textrm{PSNR}_{c\rightarrow s}$ $\pm 0.00$–$0.01\,\textrm{dB}$), evidencing highly consistent behavior across images. The actual payload BPP used (e.g., 0.383 vs. 0.400; 0.989 vs. 1.000) matches target values but shows minor systematic underperformance (0.383 instead of 0.400 and 0.989 instead of 1.000), which could be a result of selection limitations or coding requirements. The embedding scheme achieves excellent capacity expansion through a minimal degradation of cover image quality while maintaining complete secret data recovery at all tested payload levels.

**Table 4:** Quality of hiding and secret recovery at various BPP values Using Secret Image Size 64*64. Entries show mean±std [margin] for $\hbox {PSNR}_{c\rightarrow s}$, $\hbox {SSIM}_{c\rightarrow s}$, $\hbox {MSE}_{c\rightarrow s}$, $\hbox {IF}_{c\rightarrow s}$, as well as $\hbox {PSNR}_{{sec}}$, $\hbox {SSIM}_{{sec}}$, and $\hbox {BPP}_{{used}}$ over N=30 images.

BPP	N	$\hbox {PSNR}_{c\rightarrow s}$	$\hbox {SSIM}_{c\rightarrow s}$	$\hbox {MSE}_{c\rightarrow s}$	$\hbox {IF}_{c\rightarrow s}$	$\hbox {PSNR}_{{sec}}$	$\hbox {SSIM}_{{sec}}$	$\hbox {BPP}_{{used}}$
0.1	30	61.39±0.01 [0.00]	1.000±0.000 [0.000]	0.0472±0.0001 [0.0001]	1.0000±0.0000 [0.0000]	inf±nan [nan]	1.000±0.000 [0.000]	0.096±0.000
0.2	30	58.28±0.01 [0.00]	1.000±0.000 [0.000]	0.0966±0.0002 [0.0001]	1.0000±0.0000 [0.0000]	inf±nan [nan]	1.000±0.000 [0.000]	0.196±0.000
0.4	30	55.35±0.00 [0.00]	0.999±0.000 [0.000]	0.1898±0.0002 [0.0001]	1.0000±0.0000 [0.0000]	inf±nan [nan]	1.000±0.000 [0.000]	0.383±0.000
1.0	30	51.21±0.00 [0.00]	0.997±0.001 [0.001]	0.4917±0.0004 [0.0002]	0.9999±0.0000 [0.0000]	inf±nan [nan]	1.000±0.000 [0.000]	0.989±0.000

Table 5 shows the proposed method achieves perfect secret reconstruction with imperceptible cover distortion when using a $128\times 128$ secret and $N=30$ test images at $\textrm{BPP}\in \{0.1,0.2,0.4,1.0\}$. The method maintains cover image quality at 61.40 dB until it reaches 51.21 dB as BPP increases from 0.1 to 1.0 while MSE increases by a factor of 10.4 from 0.0472 to 0.4917. The SSIM and IF values remain at 1.000 for all BPP settings, which shows no perceptible change in image quality. The method achieves perfect secret recovery in every test scenario as SSIM scores reach 1.000 and PSNR values reach infinity, which indicates MSE values equal zero. The reported ”inf±nan” value represents the perfect reconstruction condition, where dispersion remains undefined. The method shows minimal image-to-image variation because PSNR values from cover to stego remain constant at 0.00–0.01 dB. The actual payload BPP values match their targets but show minor systematic underperformance at 0.383, instead of 0.400, and 0.989, instead of 1.000. The results show that the method achieves good capacity growth while maintaining low cover image distortion and complete secret data recovery at all tested payload levels.

**Table 5:** Quality of hiding and secret recovery at various BPP values using a $128\times 128$ secret image. Entries show mean±std [margin] for $\hbox {PSNR}_{c\rightarrow s}$, $\hbox {SSIM}_{c\rightarrow s}$, $\hbox {MSE}_{c\rightarrow s}$, $\hbox {IF}_{c\rightarrow s}$, as well as $\hbox {PSNR}_{{sec}}$, $\hbox {SSIM}_{{sec}}$, and $\hbox {BPP}_{{used}}$ over $N=30$ images.

BPP	N	$\hbox {PSNR}_{c\rightarrow s}$	$\hbox {SSIM}_{c\rightarrow s}$	$\hbox {MSE}_{c\rightarrow s}$	$\hbox {IF}_{c\rightarrow s}$	$\hbox {PSNR}_{{sec}}$	$\hbox {SSIM}_{{sec}}$	$\hbox {BPP}_{{used}}$
0.1	30	61.40±0.01 [0.00]	1.000±0.000 [0.000]	0.0472±0.0001 [0.0000]	1.0000±0.0000 [0.0000]	inf±nan [nan]	1.000±0.000 [0.000]	0.096±0.000
0.2	30	58.28±0.01 [0.00]	1.000±0.000 [0.000]	0.0966±0.0002 [0.0001]	1.0000±0.0000 [0.0000]	inf±nan [nan]	1.000±0.000 [0.000]	0.196±0.000
0.4	30	55.35±0.01 [0.00]	1.000±0.000 [0.000]	0.1898±0.0003 [0.0001]	1.0000±0.0000 [0.0000]	inf±nan [nan]	1.000±0.000 [0.000]	0.383±0.000
1.0	30	51.21±0.00 [0.00]	0.997±0.001 [0.001]	0.4917±0.0005 [0.0002]	0.9999±0.0000 [0.0000]	inf±nan [nan]	1.000±0.000 [0.000]	0.989±0.000

Table 6 shows the system uses a $512\times 512$ secret image with $N=30$ test images to achieve imperceptible cover distortion while recovering the secret exactly at all $\textrm{BPP}$ values from 0.1 to 1.0. The cover-to-stego PSNR value decreases from 61.39 dB to 51.21 dB when BPP increases, yet the SSIM and IF values remain at 1.000 for all rates, which shows no perceptible change. The system achieves perfect secret reconstruction in all test scenarios, producing SSIM values of 1.000 and PSNR values of infinity, which indicates MSE values of 0. The system maintains consistent behavior, showing minimal image-to-image variation in its results (e.g., $\textrm{PSNR}_{c\rightarrow s}$ values range from 0.00 dB to 0.01 dB). The system achieves its target payload rates through a small amount of systematic underfill that results in values of 0.383 instead of 0.400 and 0.989 instead of 1.000. The results show that the proposed model achieves good capacity expansion while maintaining low cover distortion and complete secret recovery at all tested payload levels.

**Table 6:** Quality of hiding and secret recovery at various BPP values using a $512\times 512$ secret image. Entries show mean±std [margin] for $\hbox {PSNR}_{c\rightarrow s}$, $\hbox {SSIM}_{c\rightarrow s}$, $\hbox {MSE}_{c\rightarrow s}$, $\hbox {IF}_{c\rightarrow s}$, as well as $\hbox {PSNR}_{{sec}}$, $\hbox {SSIM}_{{sec}}$, and $\hbox {BPP}_{{used}}$ over $N=30$ images.

BPP	N	$\hbox {PSNR}_{c\rightarrow s}$	$\hbox {SSIM}_{c\rightarrow s}$	$\hbox {MSE}_{c\rightarrow s}$	$\hbox {IF}_{c\rightarrow s}$	$\hbox {PSNR}_{{sec}}$	$\hbox {SSIM}_{{sec}}$	$\hbox {BPP}_{{used}}$
0.1	30	61.39±0.01 [0.01]	1.000±0.000 [0.000]	0.0472±0.0002 [0.0001]	1.0000±0.0000 [0.0000]	inf±nan [nan]	1.000±0.000 [0.000]	0.096±0.000
0.2	30	58.28±0.01 [0.00]	1.000±0.000 [0.000]	0.0966±0.0002 [0.0001]	1.0000±0.0000 [0.0000]	inf±nan [nan]	1.000±0.000 [0.000]	0.196±0.000
0.4	30	55.35±0.01 [0.00]	1.000±0.000 [0.000]	0.1899±0.0002 [0.0001]	1.0000±0.0000 [0.0000]	inf±nan [nan]	1.000±0.000 [0.000]	0.383±0.000
1.0	30	51.21±0.00 [0.00]	0.997±0.001 [0.001]	0.4918±0.0004 [0.0001]	0.9999±0.0000 [0.0000]	inf±nan [nan]	1.000±0.000 [0.000]	0.989±0.000

### Security analysis

We evaluate our proposed steganographic system, which includes both DL and statistical steganalysis methods. The CNN-based models Xu-Net and Ye-Net detect embedding traces through DL methods, where Xu-Net uses high-pass features and Ye-Net uses attention mechanisms for efficient detection. RS Steganalysis uses a traditional statistical method that detects LSB modifications through pixel-flipping patterns. The system receives a complete robustness evaluation through this combination of modern and traditional steganalysis techniques^39–41^.

#### Xu-Net-analysis

The Xu-Net operates as a CNN that focuses on steganalysis to identify both cover and stego images. The Xu-Net architecture implements an encoder-decoder design similar to U-Net to extract deep features and generate prediction maps for classification tasks. The training process of Xu-Net starts from scratch without pretraining as we use content-matched pairs of BOSSbase images (each cover image has its corresponding stego version) with 160 training pairs and 20 validation and test pairs that result from a deterministic split. In this section, we test and analyze the tradeoff between the payload and security at different BPP values (0.1, 0.2, 0.4, and 1.0).

**Fig. 5:**
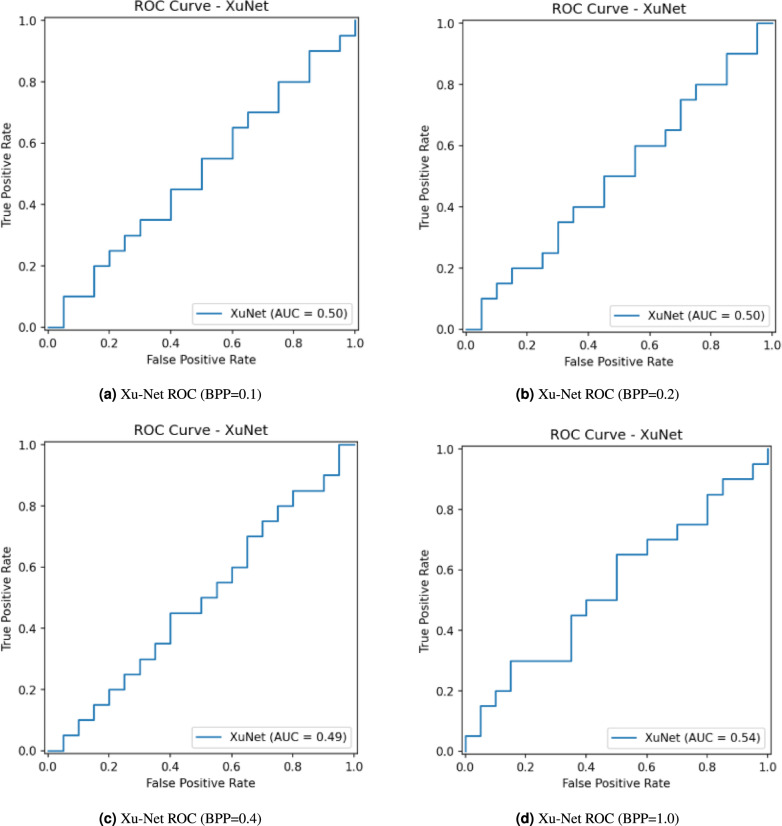
Xu-Net ROC at different BPPs with PSO.

The Receiver Operating Characteristic (ROC) analysis in Fig. 5 shows that Xu-Net achieves random performance in cover–stego discrimination for payloads of 0.1, 0.2, 0.4, and 1.0 BPP with AUC values of 0.50, 0.50, 0.49, and 0.54, respectively. The operating curves maintain their position near the chance diagonal in all cases, which indicates a 50% equal-error rate and no meaningful discrimination ability except for a small and insufficient improvement at 1.0 BPP (AUC= 0.54 < 0.6, which is a typical threshold for weak discrimination). The network achieves chance-level performance at all examined payload levels except for a minimal improvement at 1.0 BPP, which results in an AUC value of 0.54 that is below the typical 0.6 threshold for weak discrimination.

**Fig. 6:**
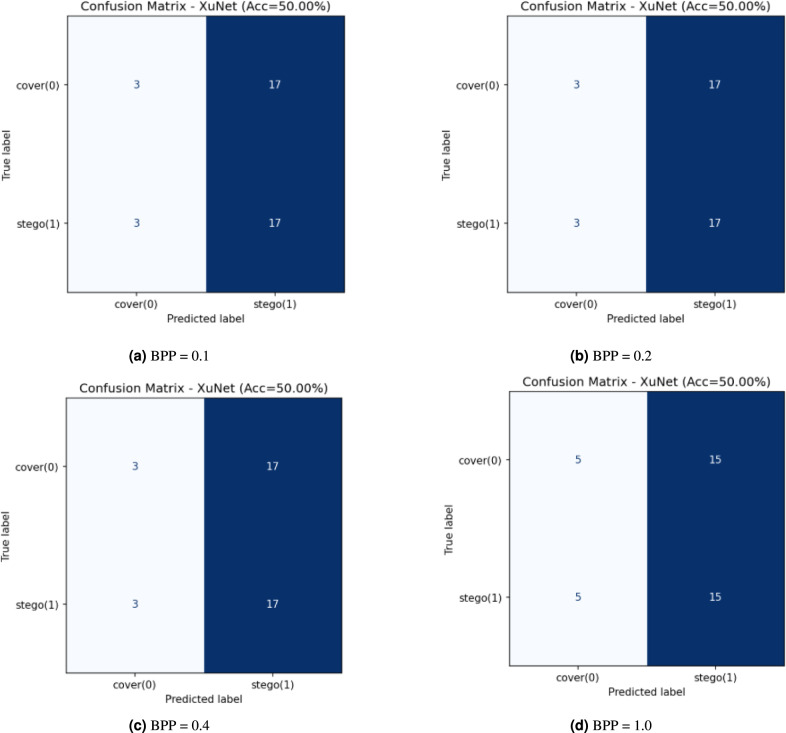
Xu-Net confusion matrices at different BPPs with PSO.

The confusion matrices in Fig. 6 show Xu-Net performance across four different payloads (BPP = 0.1, 0.2, 0.4, and 1.0) when testing on a balanced cover–stego dataset. The classifier shows steer-bias at the three lower payload levels because it predicts the stego label for most inputs from both classes (e.g., 3/40 covers are misclassified as cover while 37/40 are misclassified as stego and the same pattern occurs for true stegos). The high True Positive Rate (TPR) $\textrm{TPR} \approx 0.93$ matches the high False Positive Rate (FPR) $\textrm{FPR} \approx 0.93$, which results in a 50% accuracy rate when the classes are balanced. The BPP = 1.0 threshold shows a minor reduction in bias, but the accuracy stays at chance levels according to both the ROC/AUC analysis with $\textrm{AUC} \approx 0.54$ and the confusion matrix results. The current thresholding of Xu-Net fails to provide reliable discrimination for these payloads because it sacrifices all specificity to achieve higher sensitivity at low and mid BPP levels and never reaches a useful operating point. The detector fails to detect stego artifacts because the embedding/encryption pipeline generates difficult-to-exploit artifacts, which results in weak separability that appears only through AUC ranking rather than fixed-threshold confusion matrix counts.

The statistical results in Table 7 demonstrate Xu-Net’s cover–stego discrimination ability for different payload sizes but show no significant performance difference from random chance level. The test set balance produces fixed accuracy results of 50.00% for all BPPs because the default decision threshold produces no class separation. The threshold-free metric shows that the AUC values at 0.1 and 0.2 BPP are equal to 0.5025, which indicates random guessing performance. The AUC value of 0.4900 at 0.4 BPP falls below chance, while the AUC value of 0.5425 at 1.0 BPP shows weak separability that falls below typical detection thresholds ($\textrm{AUC}\!\ge \!0.6$ for weak and $\textrm{AUC}\!\ge \!0.7$ for moderate).

**Table 7:** Performance of Xu-Net on cover and stego datasets at different BPP values in terms of accuracy and AUC with PSO.

Dataset	Accuracy	AUC
Cover_Stego_0.1BPP	50.00%	0.5025
Cover_Stego_0.2BPP	50.00%	0.5025
Cover_Stego_0.4BPP	50.00%	0.4900
Cover_Stego_1.0BPP	50.00%	0.5425

**Fig. 7:**
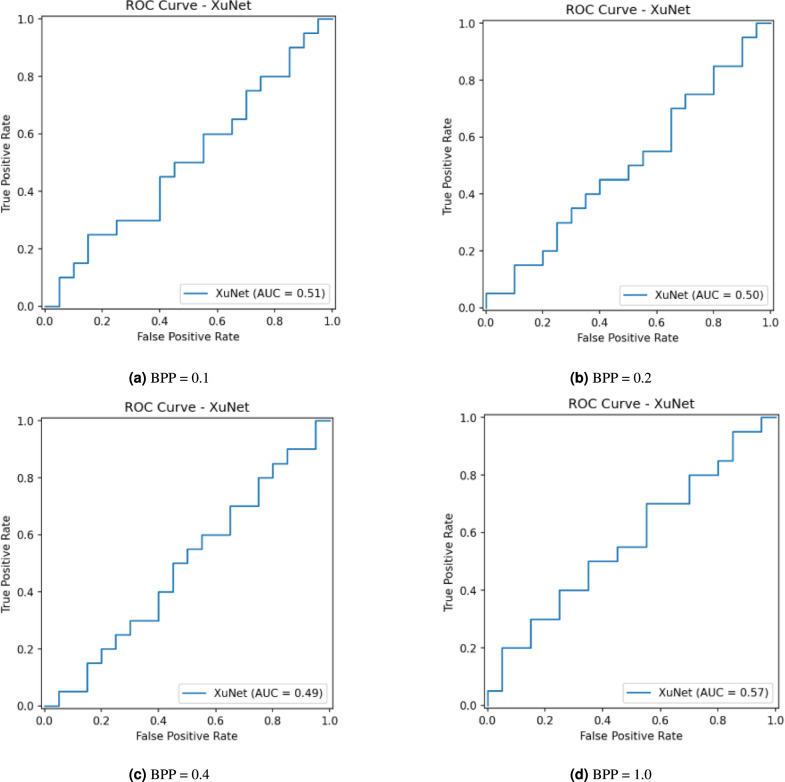
Xu-Net ROC at different BPPs without PSO.

Figure 7 shows the ROC analysis section presents the ROC curves for Xu-Net when BPP equals 0.1, 0.2, 0.4, and 1.0 (without PSO). The ROC curves follow the diagonal line for the first three payloads with the AUC values remaining at 0.51 (BPP = 0.1), 0.50 (BPP = 0.2), and 0.49 (BPP = 0.4), which indicates random-level discrimination between cover and stego images. The AUC value reaches 0.57 at BPP = 1.0, but the improvement remains minimal and fails to create an operating region that outperforms random guessing. The ROC results match the confusion matrices, which present high false-positive rates and accuracy levels around 50%.

**Fig. 8:**
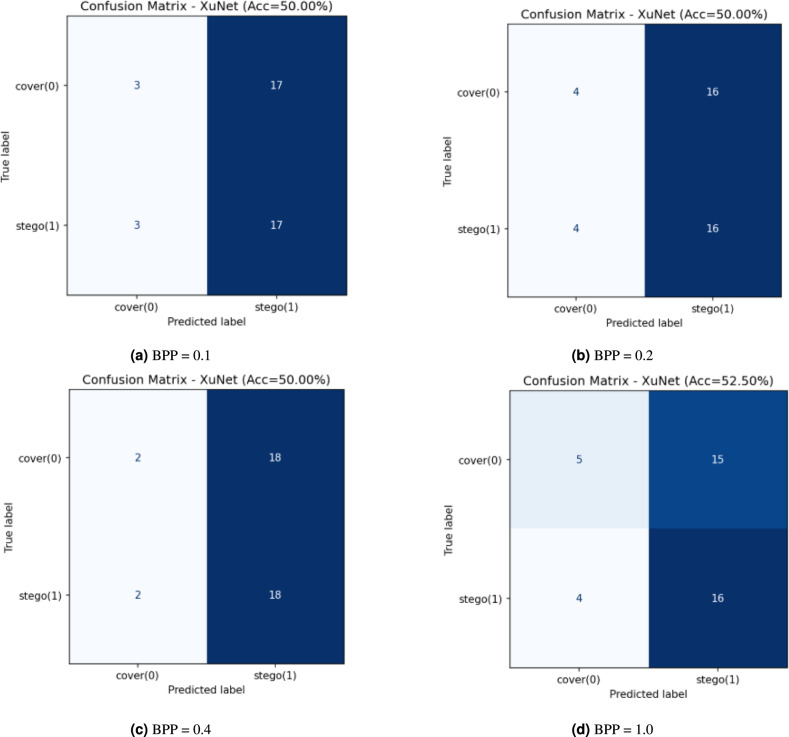
Xu-Net confusion matrices at different BPPs without PSO.

Figure 8 presents the confusion matrices of the Xu-Net models when operating with different payload values. The network maintains a consistent accuracy level of 50–52.5% across all payload values but fails to distinguish between our method’s stegos and covers. The classifier demonstrates a continuous preference for identifying stego images. The classifier achieves a high TPR of 0.8–0.9 for stego images yet produces very low True Negative Rates (TNRs) of 0.1–0.25 for cover images, which results in numerous false positives and maintains an overall accuracy of 50%. The TPR shows a minor improvement at BPP = 1.0 while the model maintains poor specificity levels.

Table 8 shows Xu-Net performance results for different payloads (without PSO), which demonstrates near-chance discrimination at low–medium rates. The classifier shows no ability to distinguish between cover and stego data because it maintains 50.00% accuracy at 0.1, 0.2, and 0.4 BPP while AUC values remain at 0.5075, 0.5000, and 0.4900. The classifier shows a slight improvement at 1.0 BPP with 52.50% accuracy and AUC of 0.57, yet it does not achieve effective separation.

**Table 8:** Xu-Net performance across payloads without PSO.

Dataset	Accuracy	AUC
Cover_Stego_0.1BPP	50.00%	0.5075
Cover_Stego_0.2BPP	50.00%	0.5000
Cover_Stego_0.4BPP	50.00%	0.4900
Cover_Stego_1.0BPP	52.50%	0.5700

#### Ye-Net-analysis

The spatial-domain image steganalyzer Ye-Net functions through its implementation of a Convolutional Neural Network (CNN) structure. The system starts by performing fixed high-pass residual preprocessing to decrease image content visibility while enhancing embedding artifacts before it runs Truncated Linear Unit (TLU) for dynamic range restriction and multiple Conv-BN (and pooling) blocks to extract discriminative residual features and a linear/softmax classifier for cover vs. stego detection. The model receives no pretraining because we train Ye-Net from scratch using content-matched pairs of images (each cover image has its corresponding stego version), which we split into 160 training pairs, 20 validation pairs, and 20 testing pairs through a deterministic method.

**Fig. 9:**
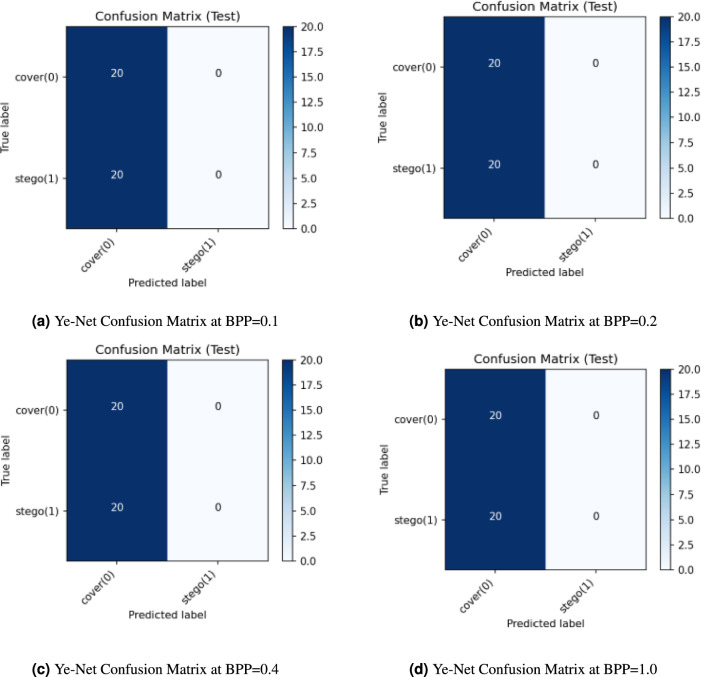
Ye-Net Confusion Matrices at different BPP values with PSO.

The classification results from Ye-Net appear in Fig. 9, which shows performance at different embedding rates (BPP = 0.1, 0.2, 0.4, and 1.0). The model failed to detect any stego images in all payload tests because it incorrectly identified every stego image as a cover image, which resulted in zero detection accuracy for the stego class. The model correctly identified all 20 cover images at each BPP value but incorrectly classified all 20 stego images as cover images. The current configuration of Ye-Net shows a strong preference for cover class predictions, which prevents it from detecting hidden payloads at any embedding rate.

**Fig. 10:**
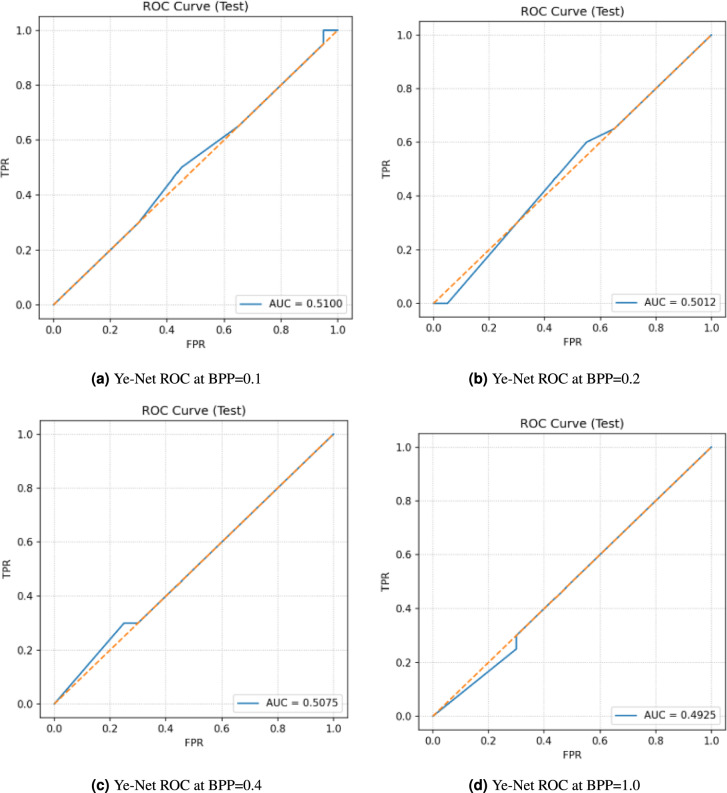
Ye-Net ROC Curves at different BPP values with PSO.

The ROC curves of Ye-Net at different embedding rates (BPP = 0.1, 0.2, 0.4, and 1.0) in Fig. 10 demonstrate that the model shows very poor discriminative power for steganalysis tasks. The ROC curves maintain a diagonal pattern throughout all tested payload sizes, which indicates the model performs at a random classification level. The AUC values stay near 0.5 with results of 0.5100, 0.5012, 0.5075, and 0.4925 when BPP equals 0.1, 0.2, 0.4, and 1.0, respectively. The results demonstrate that Ye-Net lacks the ability to identify stego images from cover images at every embedding rate.

The model achieves performance results for different embedding rates (BPP = 0.1, 0.2, 0.4, and 1.0), as shown on Table 9, which shows accuracy and AUC values. The model demonstrates consistent 50% accuracy performance for all tested payloads because it makes random predictions between cover and stego images. The AUC values show minimal changes between 0.4925 and 0.5100 across all embedding rates. The model demonstrates no ability to detect discriminative features between cover and stego images at any embedding payload level. The fixed 50% accuracy rate, combined with AUC values near chance level, demonstrates the proposed system’s restricted capabilities. The inability of Ye-Net to detect cover and stego images indicates successful information concealment through steganography because the embedding process created no detectable traces of hidden data.

**Table 9:** Performance of Ye-Net on cover and stego datasets at different BPP values in terms of accuracy and AUC with PSO.

Dataset	Accuracy	AUC
Cover_Stego_0.1BPP	50.00%	0.5100
Cover_Stego_0.2BPP	50.00%	0.5012
Cover_Stego_0.4BPP	50.00%	0.5075
Cover_Stego_1.0BPP	50.00%	0.4925

**Fig. 11:**
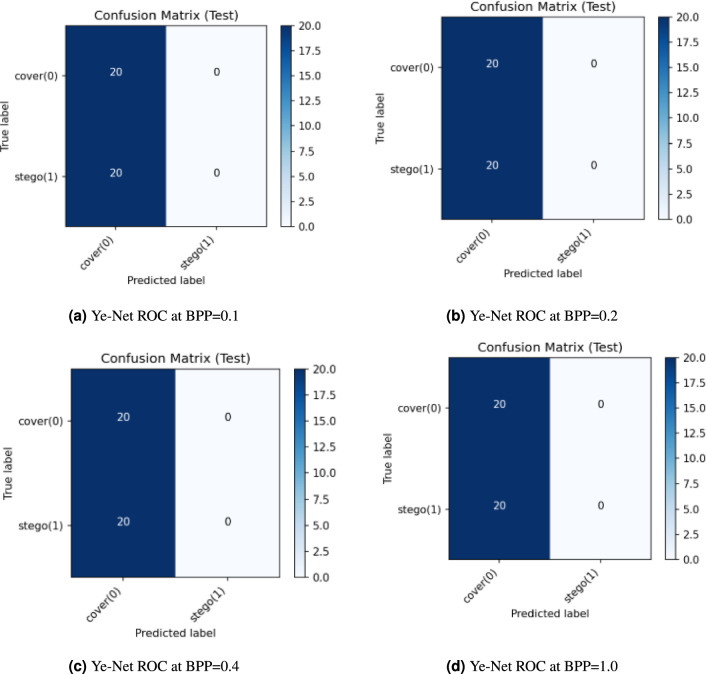
Ye-Net confusion matrix at different BPP values without PSO.

Figure 11 shows the confusion matrices of **Ye-Net** for a balanced test set containing 20 cover images and 20 stego images that appear at different payload levels of BPP equal to 0.1, 0.2, 0.4, and 1.0. The network identifies all test samples as cover images because it correctly identifies all cover images (TN = 20 and FP = 0) but incorrectly identifies all stego images (TP = 0 and FN = 20). The network achieves 50% accuracy because it misclassifies all stego images while correctly identifying all cover images. The stego class achieves 0% recall (TPR) and 100% specificity (True Negative Rate (TNR)) while precision and F1 scores for the stego class remain at 0. The balanced accuracy reaches 0.5 because the dataset is balanced, which represents performance equivalent to that of random guessing. The model maintains its ”always-cover” classification behavior regardless of payload size because it fails to detect steganographic content.

**Fig. 12:**
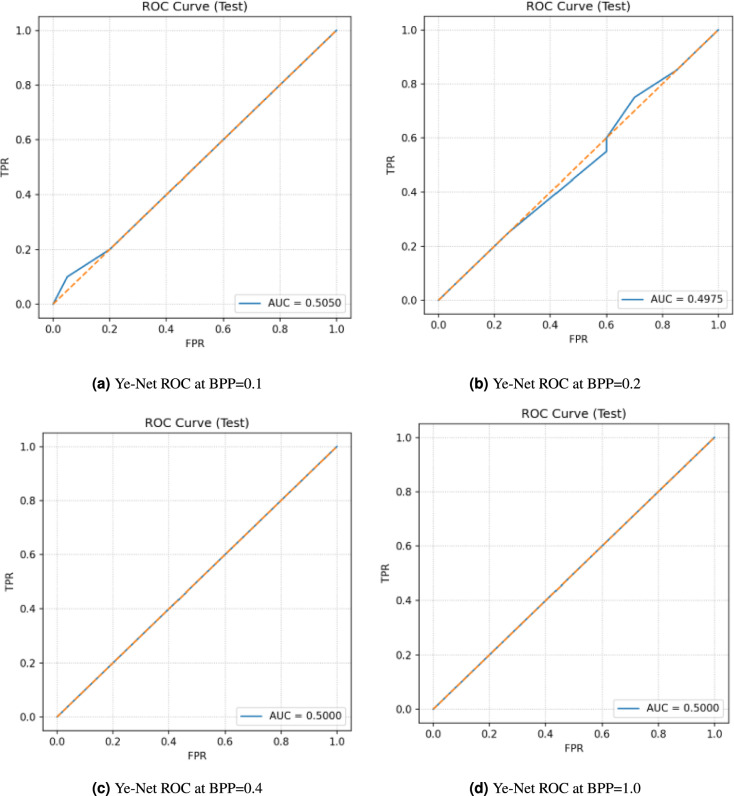
Ye-Net ROC curves at different BPP values without PSO.

The ROC curves of **Ye-Net** for a balanced test set with four different payloads at BPP values from 0.1 to 1.0 are shown in Fig. 12. The empirical ROC curve shows no deviation from the chance diagonal in all panels because the AUC values remain at 0.5050, 0.4975, 0.5000, and 0.5000. The lack of curvature toward the upper-left corner in the ROC curve shows that the cover and stego scores have no practical distinction, which makes threshold tuning ineffective because the underlying score distributions appear identical. The AUC values remain constant across different BPP settings, which indicates that the proposed model shows no response to changes in payload size.

The performance results of **Ye-Net** during testing are shown in Table 10 for BPP values of 0.1, 0.2, 0.4, and 1.0. The AUC values remain at chance level (0.5050, 0.4975, 0.5000, 0.5000) while accuracy stays constant at 50.00% for all testing conditions. The combination of metrics in a balanced cover/stego evaluation shows chance-level discrimination because threshold tuning fails to produce meaningful results due to non-separable score distributions. The model shows no response to payload size variations because both accuracy and AUC remain constant across different BPP values.

**Table 10:** Ye-Net performance on balanced test sets at different payloads without PSO.

Dataset	Accuracy	AUC
Cover_Stego_0.1BPP	50.00%	0.5050
Cover_Stego_0.2BPP	50.00%	0.4975
Cover_Stego_0.4BPP	50.00%	0.5000
Cover_Stego_1.0BPP	50.00%	0.5000

#### RS-analysis

The blind spatial-domain steganalysis technique RS analysis, specifically targets LSB replacement methods. The image is divided into non-overlapping pixel groups *G*; a simple discriminating function *f*(*G*) is evaluated before and after applying LSB flipping via the mask *M* and its inverse $-M$, yielding $F_M(G)$. The method RS works efficiently for grayscale and color images with LSB replacement, but its detection power weakens when dealing with LSB matching and textured images and intense image processing operations.

**Fig. 13:**
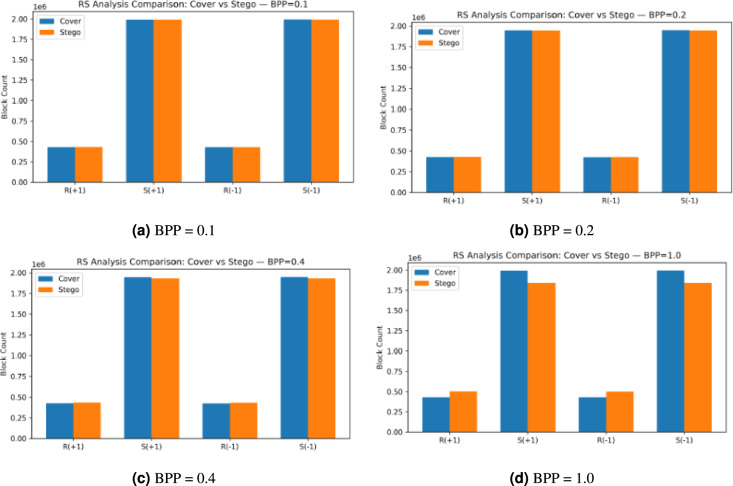
RS analysis comparison between cover and stego images at different embedding rates (BPP = 0.1, 0.2, 0.4, and 1.0) with PSO.

The results from RS analysis of cover and stego images at different embedding rates (BPP of 0.1, 0.2, 0.4, and 1.0) are shown in Fig. 13. The histograms show the block count distributions for the four RS groups, which include $R(+1),\ S(+1),\ R(-1),\ \text {and}\ S(-1)$. The RS method detects statistical changes from data embedding through noticeable distribution differences between stego images and their corresponding cover images at embedding rates between 0.1 and 0.4. The RS analysis shows reduced sensitivity when embedding rates reach their maximum (BPP = 1). The method becomes less effective at detecting hidden data because of saturation effects that occur when modifications exceed certain limits. The results demonstrate that RS analysis works well for detecting hidden data at typical payload levels, which proves that our proposed method can maintain high imperceptibility, PC, and security. However, the performance of our proposed model decreases when embedding rates become too high..

The RS Analysis summary, as shown in Table 11, demonstrates how the RS Steganalysis technique detects statistical changes that occur when data embedding happens at various BPP rates. The RS values at BPP equal to 0.1, 0.2, and 0.4 show minimal standard deviation while maintaining a stable average value of $\approx 0.66$. The local image structural patterns remain intact when data embedding occurs at these specific rates. The maximum BPP rate of 1.0 leads to a decrease in mean RS value to $\approx 0.59$. The heavy pixel modification during the maximum embedding rate causes the statistical difference between Regular and Singular groups to become less distinguishable, which results in a saturation effect. The RS analysis shows a non-linear response pattern because it detects moderate embedding rates effectively, but fails to provide reliable results when dealing with high payload amounts. The results of this research indicate that RS analysis works best for detecting moderate embedding strengths but needs additional detection models for better performance at high embedding capacities.

**Table 11:** RS analysis results at different embedding rates (BPP) with PSO.

BPP	Mean RS	Std. Dev.	N (images)
0.1	0.6651	0.1735	200
0.2	0.6633	0.1719	200
0.4	0.6656	0.1667	200
1.0	0.5921	0.1462	200

**Fig. 14:**
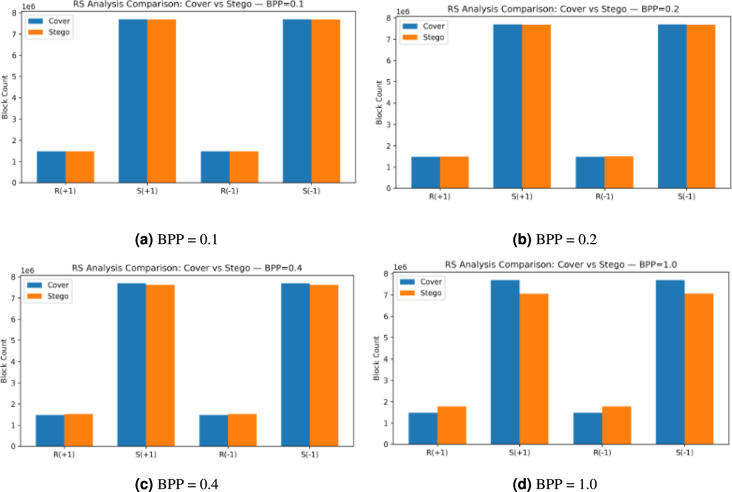
RS analysis comparison between cover and stego images at different embedding rates (BPP = 0.1, 0.2, 0.4, and 1.0) without PSO.

Figure 14 shows the four RS components R(+1), S(+1), R(-1) rates BPP = 0.1, 0.2, 0.4, and S(-1) between cover (blue) and stego (orange) images at different embedding 1.0. The bar heights of cover and stego images show no difference for all components at the low payloads of 0.1 and 0.2 BPP. The RS analysis shows no detectable difference between cover and stego images when the payload is 0.1 and 0.2 BPP. The stego images show the expected changes in pixel-pair groups under ±1 perturbations at 0.4 BPP as the regular counts decrease and singular counts increase, which produces a clear distinction between cover and stego images. The RS method shows no detectable changes. The local pair statistics undergo significant changes during the embedding process at 0.1 BPP, which indicates the ability to detect changes at low embedding rates.

The RS analysis for stego datasets without PSO is presented in Table 12, which shows that the RS statistic maintains stability at low to moderate payload levels when BPP equals 0.1, 0.2 or 0.4 with N set to 200. The RS statistic shows no detectable structural changes because the means stay at 0.66 and the standard deviation remains at 0.17 for 0.1, 0.2, and 0.4 BPP, and at 0.1462 for 1.0 BPP, which indicates that heavy pixel modifications have reached saturation and made Regular and Singular group separation less effective. The RS shows no reaction to changes in the values. The BPP value of 1.0 leads to a mean value in the 0.1 to 0.4 BPP range, but starts to detect changes at 1.0 BPP, which indicates that the 0.2 to 0.4 BPP range provides the best balance between data capacity and detectability.

**Table 12:** RS statistics for stego datasets at different payloads without PSO.

Dataset	BPP	RS (mean ± std)	N
stegos_BPP_0_1	0.1	$0.6651 \pm 0.1735$	200
stegos_BPP_0_2	0.2	$0.6634 \pm 0.1719$	200
stegos_BPP_0_4	0.4	$0.6559 \pm 0.1663$	200
stegos_BPP_1_0	1.0	$0.5906 \pm 0.1466$	200

To explain the security vs. payload trade-off, we present the research results demonstrating that spatial steganography requires users to choose between higher security and increased data capacity. The visual quality decreases progressively when payload rates rise from low to high while the RS signal strength increases, which makes statistical detection more probable. The practical operating range for our system is between 0.2 and 0.4 BPP because it maintains excellent visual quality and produces a relatively low RS response. The 1.0 BPP setting functions as a testing condition rather than a suggested operational point, as it provides excessive detectability at the cost of increased capacity. The system includes a multi-objective control that enables users to adjust distortion and detectability costs while using adaptive payload distribution based on the fused map and restricted coding to reduce pixel modifications per embedded bit. The system offers defined thresholds, which enable users to select their payload level based on security and quality needs for specific applications through a reproducible method.

The baseline system uses LSB replacement for moderate-to-high payloads (0.1 BPP up to 1.0 BPP), yet our adaptive selection channel operates through fused entropy. RS analysis detection capability becomes possible because half of the embedded LSBs experience flipping during these conditions, which creates a significant RS imbalance. The selection channel operates deterministically, which makes it vulnerable to RS analysis, while our PSO objective focuses on minimizing MSE instead of RS evidence, thus failing to suppress the RS signal. The system uses four methods to reduce detectability: (i) it switches from LSB replacement to LSB matching with $\pm 1$ values, (ii) it applies a keyed permutation to ranked indices for breaking spatial patterns, (iii) it uses matrix/wet-paper (or STC) coding to decrease the number of modified samples at constant payload, and (iv) it conducts evaluations at reduced BPP levels. The implemented modifications decrease the absolute difference between $R_M$ and $S_M$ and between $R_{-M}$ and $S_{-M}$ while making the RS payload estimate $\hat{p}$ approach zero without compromising cover quality (PSNR/SSIM).

The proposed method shows high resistance to CNN-based detectors, yet RS analysis reveals detectable statistical patterns as shown in Table 11. The spatial-domain scheme produces this behavior because flipping operations at the least-significant bit level affects the regular–singular block statistics, which RS uses for analysis even when the fused map guides the payload to rich areas. The optimizer will receive an RS-aware cost function to prevent candidate embeddings that enhance RS discrimination signals from being selected. The evaluation will include a transform-domain version (DCT/DWT), which uses the same guidance map to process robust coefficients and a differentiable RS surrogate for adversarial training to teach the encoder suppression of RS indicators.

### Comparing with previous work

As shown by the findings presented on Table 13, the proposed method uses BPP units for reporting payload, whereas JPEG-domain baselines use bpnzAC units; this makes direct comparison between them challenging. The proposed method achieves superior performance in terms of capacity-security-imperceptibility trade-offs because it reaches MSE = 0, PSNR = 51dB, SSIM = 0.9971, and IF = 0.99 at 1 BPP (262144 bits on a 512$\times$512 cover) while maintaining perceptual quality equivalent to previous work that operates at 0.4 BPP or 0.5 bpnzAC. The proposed method demonstrates security against Xu-Net, Ye-Net, and RS while maintaining high PC, which extends the Pareto front. The results solve the long-standing problem of needing to reduce payload to achieve both visual imperceptibility and detection resistance because this method enables high payload, high fidelity, and strong steganalysis resistance, simultaneously. Therefore, our proposed method achieves three major advancements through its ability to increase usable capacity by a large factor without degrading perception, its alignment with modern CNN steganalyzers, and its demonstration of learned constraints that produce minimal modification effects. The method requires additional testing for channel distortion resistance through BER measurements at JPEG Q = 65-95 levels and P_e/AUC evaluations against SRM/EC, SRNet, Ye-Net, and Yedroudj-Net detectors to establish absolute superiority. Our proposed method outperforms previous steganographic methods according to the reported evaluation metrics^42^.

**Table 13:** Comparison between our work and related works based on USC-SIPI dataset.

Paper	Dataset	Imperceptibility	Capacity	Security	Robustness
^18^	BOSSbase	PSNR>35	0.05-0.5 bpnzAC	DCTR=secure, GFR=secure	Error rate from JPEG compression Q 75,Q95
^17^	BOSSbase	PSNR>50	0.01-0.1 bpnzAC	DCTR=secure,GFR=secure	Error rate from JPEG compression Q 65,Q75,Q 85
^19^	BOSSbase	PSNR>52	0.05-0.3 bpnzAC	DCTR=secure,GFR=secure	Error rate from JPEG compression Q 75,Q85,Q 90
^20^	BOSSbase	NA	0.1-0.5 bpnzAC	DCTR=secure, SR net=secure	Error rate from JPEG compression Q 70
^23^	BOSSbase	NA	NA	Achieved the lowest detection accuracy across five steganalyzers (SRM, Deng-Net, SRNet, Yedroudj-Net, Ye-Net), improving security by up to 5.39% over prior methods	NA
^21^	BOSSbase	PSNR=50-55, SSIM=0.9991	655,144 pixels,BPP=0.1-0.4	XU-Net=NA, Ye-Net=NA,RS=NA	NA
^22^	BOSSbase	PSNR=41.2, SSIM=0.9821	BPP=0.1-0.4	SR-Net=Secure, Ye-Net=NA,RS=NA	NA
Proposed Work	BOSSbase	MSE=0.0470,PSNR=61.39, SSIM=1.0,IF=0.999998	262,144 pixels,BPP=0.096-0.989	XU-Net=Secure, Ye-Net=Secure,RS=Secure	NA

## Future work

The following research directions are proposed as future extensions and were not investigated within the scope of the current study. Grayscale datasets were selected to enable controlled experimentation and fair comparison with established steganography benchmarks. Therefore, future research directions should concentrate on implementing the proposed system for color images and video data to increase its practical application. The embedding efficiency of the system can be enhanced by implementing adaptive fusion strategies and by using alternative optimization algorithms or hybrid metaheuristics like Genetic Algorithm (GA), Gray Wolf Optimization (GWO), ACO, and others. The system’s robustness against DL-based steganalysis methods can be enhanced by incorporating adversarial training using GAN versions. Real-world deployment scenarios, such as secure image sharing and telemedicine may offer valuable directions for evaluation and refinement. These scenarios are currently considered at a conceptual level and have not yet been validated in operational environments.

Robustness against lossy JPEG recompression was not evaluated in the current study and remains an open limitation. The channel-aware protocol includes two operational modes, which involve (a) stego-last, where stego images undergo standard JPEG compression at different Quality Factors (QF) from 95 to 30 QF, and (b) channel-processed-cover embedding, which embeds data into pre-compressed images to detect channel errors. A systematic robustness evaluation across different payload ranges was not conducted in the current experiments. Future study will also evaluate each condition and payload range from 0.1 to 0.4 BPP by measuring (a) secret extraction accuracy through BER and SSIM/PSNR comparison between the secret data and recovered information, (b) the perceptual quality of recompressed stego images through PSNR/SSIM analysis relative to the original cover image, (c) the amount of effective payload data maintained, and (d) the detectability of the steganographic method using RS steagnalysis, CNN steganalyzers like Ye-Net and Xu-Net on channel-matched datasets (AUC/ROC). Detectable artifacts under RS analysis were observed only under specific payload and channel conditions.

The proposed system offers operational advantages for contexts with secure communication needs, like telemedicine and confidential image exchange, as it enables real-time imperceptible data transmission of sensitive information. However, computational complexity, latency, and resource overhead were not quantitatively evaluated in the current study. This research study presents promising results, yet it has several limitations. The system evaluation took place with only grayscale images from BOSSbase and USC-SIPI datasets, which limits generalizability to work with color images and video content in practical scenarios. The use of only two datasets may introduce dataset bias, and broader dataset validation is required. Therefore, additional datasets are required to confirm generalizability. The system proved resistant to both statistical and CNN-based steganalysis methods (XU-Net and Ye-Net), yet it still shows detectable artifacts when subjected to classical RS analysis under specific conditions. These artifacts were observed only under particular payload and channel configurations rather than consistently across all test cases. The optimization process used PSO with fixed parameters, but further research with different or combined metaheuristics like GA, GWO, and ACO could lead to better performance results. The PSO optimizer was implemented with fixed parameters, which may limit optimal convergence. This indicates that parameter adaptation and optimizer hybridization were not explored in this work.

The current testing on grayscale images restricts our model from being used directly for color images. Therefore, future studies should be conducted in the color space by embedding information into chroma channels while maintaining luminance values, and should learn separate attention mechanisms for each color channel while enforcing consistency between color channels (RGB). The future models should receive three-channel input data with CBAM attention fusion to assist learning through color-aware perceptual loss functions. These future efforts can demonstrate performance tradeoffs between capacity, fidelity, and detectability, while also incorporating additional tests on color image and compressed video datasets against current steganalysis benchmarks for color and video content.

## Conclusions

This paper introduces an advanced image framework that combines fused map creation with Blowfish encryption and PSO-based optimization to achieve better security alongside improved imperceptibility and data recovery capabilities. The system uses entropy and Laplacian-based noise features to create a fused attention map, which identifies sensitive regions for embedding while PSO optimized positions to achieve maximum PSNR and minimum BER. The experimental results show that the system maintains high visual quality through PSNR values of 61.39 dB and reached SSIM of 1.0 while achieving zero BER and infinity PSNR for perfect secret image recovery. The proposed method proved highly resistant to steganalysis attacks during the evaluation process, as demonstrated by the fact that the XU-Net and Ye-Net CNN-based classifiers demonstrated poor ability to identify stego images from cover images with AUC scores remaining below 0.49-0.57. RS analysis showed minimal statistical detectability, which confirms the system’s high level of undetectability. The embedding and extraction procedures operated at real-time speeds, with execution times under 0.25 seconds, which makes this approach suitable for secure communication applications. The combination of fused-map guidance with evolutionary optimization and lightweight encryption creates a major advancement in the development of secure and intelligent steganographic frameworks.

## Data Availability

The experimental code can be accessed through the following link: 10.6084/m9.figshare.29651585.v2.
